# Induction of GADD34 Is Necessary for dsRNA-Dependent Interferon-β Production and Participates in the Control of Chikungunya Virus Infection

**DOI:** 10.1371/journal.ppat.1002708

**Published:** 2012-05-17

**Authors:** Giovanna Clavarino, Nuno Cláudio, Thérèse Couderc, Alexandre Dalet, Delphine Judith, Voahirana Camosseto, Enrico K. Schmidt, Till Wenger, Marc Lecuit, Evelina Gatti, Philippe Pierre

**Affiliations:** 1 Centre d'Immunologie de Marseille-Luminy, UM2, Aix-Marseille Université, Marseille, France; 2 INSERM, U1104, Marseille, France; 3 CNRS, UMR 7280, Marseille, France; 4 Institut Pasteur, ‘Microbes and host barriers’ Group, Paris, France; 5 Inserm, Equipe avenir U604, Paris, France; 6 Université Paris Descartes, Hôpital Necker-Enfants malades, Service des Maladies Infectieuses et Tropicales, Assistance Publique-Hôpitaux de Paris, Paris, France; University of North Carolina at Chapel Hill, United States of America

## Abstract

Nucleic acid sensing by cells is a key feature of antiviral responses, which generally result in type-I Interferon production and tissue protection. However, detection of double-stranded RNAs in virus-infected cells promotes two concomitant and apparently conflicting events. The dsRNA-dependent protein kinase (PKR) phosphorylates translation initiation factor 2-alpha (eIF2α) and inhibits protein synthesis, whereas cytosolic DExD/H box RNA helicases induce expression of type I-IFN and other cytokines. We demonstrate that the phosphatase-1 cofactor, growth arrest and DNA damage-inducible protein 34 (GADD34/Ppp1r15a), an important component of the unfolded protein response (UPR), is absolutely required for type I-IFN and IL-6 production by mouse embryonic fibroblasts (MEFs) in response to dsRNA. GADD34 expression in MEFs is dependent on PKR activation, linking cytosolic microbial sensing with the ATF4 branch of the UPR. The importance of this link for anti-viral immunity is underlined by the extreme susceptibility of GADD34-deficient fibroblasts and neonate mice to Chikungunya virus infection.

## Author Summary

Nucleic acids detection by multiple molecular sensors results in type-I interferon production, which protects cells and tissues from viral infections. At the intracellular level, the detection of double-stranded RNA by one of these sensors, the dsRNA-dependent protein kinase also leads to the profound inhibition of protein synthesis. We describe here that the inducible phosphatase 1 co-factor Ppp1r15a/GADD34, a well known player in the endoplasmic reticulum unfolded protein response (UPR), is activated during double-stranded RNA detection and is absolutely necessary to allow cytokine production in cells exposed to poly I:C or Chikungunya virus. Our data shows that the cellular response to nucleic acids can reveal unanticipated connections between innate immunity and fundamental stress pathways, such as the ATF4 branch of the UPR.

## Introduction

During their replication in host cells, RNA and DNA viruses generate RNA intermediates, which elicit antiviral responses mostly through type-I interferon (IFN) production [1], [2]. Several families of proteins are known to sense double-stranded RNA (dsRNA), including endocytic Toll-like receptor 3 (TLR3) [3], the dsRNA-dependent protein kinase (PKR) [4] and the interferon-inducible 2′-5′-oligoadenylates and endoribonuclease L system (OAS/2-5A/RNase L) [5]. Viral dsRNA and the synthetic dsRNA analog polyriboinosinic:polyribocytidylic acid (poly I:C) are also detected by different cytosolic DExD/H box RNA helicases such as the melanoma differentiation-associated gene 5 (MDA5), DDX1, DDX21, and DHX36, which, once activated, trigger indirectly the phosphorylation and the nuclear translocation of transcription factors such as IRF-3 and IRF-7, resulting predominantly in abundant type-I IFN and pro-inflammatory cytokines production by the infected cells [1], [6], [7].

Alphaviruses such as Chikungunya virus (CHIKV) are small enveloped viruses with a message-sense RNA genome, which are known to be strong inducers of type-I IFN *in vivo* [8], [9], a key response for the host to control the infection [10], [11], [12]. *In vitro*, however, response to RNA viruses is heterogeneous, since Sindbis virus (SINV), do not elicit detectable IFN-α/β production in infected of murine embryonic fibroblasts (MEFs) [13]. The specific points of blockage of type-I IFN production during infection are still not well delineated, but SINV and other alphaviruses could antagonize IFN production by shut-off of host macromolecular synthesis in infected cells [14], [15], [16]. Recently, human fibroblasts infection by CHIKV was shown to trigger abundant IFN-α/β mRNA transcription, while preventing mRNA translation and secretion of these antiviral cytokines [13], [15]. Contrasting with these reports, other groups using different CHIKV strains have observed abundant type-I IFNs release in the culture supernatants of CHIKV-infected human monocytes [17], human lung cells (MRC-5), human foreskin fibroblasts and MEFs [10]. Type-I IFN stimulation of non-hematopoietic cells has also been shown to be essential to clear infection upon CHIKV inoculation in mouse, but CHIKV was found to be a poor inducer of IFN secretion by human plasmacytoïd dendritic cells [10]. Thus, great disparities regarding alphavirus-triggered IFN responses exist between viral strains and the nature of host cells or animal models.

Once bound to their receptor on the cell surface (IFNAR), type-I IFNs activate the Janus tyrosine kinase pathway, which induces the expression of a wide spectrum of cellular genes including *Pkr* [18]. These different genes participate in the cellular defense against the viral infection. PKR is a serine–threonine kinase that binds dsRNA in its N-terminal regulatory region and induces phosphorylation of translation initiation factor 2-alpha (eIF2α) on serine 51 [19], [20], leading to protein synthesis shut-off and apoptosis. PKR has been also been shown to participate in several important signaling cascades, including the p38 and JNK pathways [21], as well as type-I IFN production [22], [23]. Inhibition of translation, IFN responses and triggering of apoptosis combine to make PKR a powerful antiviral molecule, and many viruses have evolved strategies to antagonize it [24], [25]. Interestingly, several positive RNA-strand viruses (eg. *Togaviridae* or *Picornaviridae*) have been shown to activate PKR, resulting in phosphorylation of eIF2α and host translation arrest [26], while viral mRNA could initiate translation in an eIF2-independent manner by means of a dedicated RNA structure, that stalls the scanning 40S ribosome on the initiation codon [25].

Despite the existence of these viral PKR-evading strategies, the importance of PKR for type-I IFN production has been strongly debated over the years and even considered dispensable since the discovery of the innate immunity function of the DExD/H box RNA helicases [27], [28]. However, several PKR-deficient cell types have reduced type-I IFN production in response to poly I:C [23], [29], [30], while PKR was demonstrated to be required for IFN-α/β production in response to a subset of RNA viruses including Theiler's murine encephalomyelitis, West Nile (WNV) and Semliki Forest virus (SFV), but not influenza, Newcastle disease, nor Sendai virus [31], [32], [33], [34]. These studies raise therefore the possibility that some but not all viruses induce IFN-α/β in a PKR-dependent and cell specific manner. Infection of PKR or RNAse L deficient mice demonstrated that these enzymes were not absolutely necessary for type I IFN-mediated protection from alphaviruses such as SFV or WNV, but still contributed to levels of serum IFN and clearance of infectious virus from the central nervous system [25], [35]. Similarly, deficient mice for both PKR and RNAse L showed no increase in morbidity following SINV infection, although, like during WNV infection, increased viral loads in draining lymph nodes were observed [35], [36]. These observations support a non-redundant and cell specific role for these enzymes in the amplification of type-I IFN responses to viral infection more than in their initiation [31], [32], [35]. Nevertheless, the exacerbated phenotypes observed upon alphavirus infection of mice deficient for type-I IFN receptor (IFNAR), underlines the limits of the individual contributions of PKR and RNAse L to the anti-viral resistance of adult animals [10], [35], [36].

In addition to dsRNA detection, different stress signals trigger eIF2α phosphorylation, thus attenuating mRNA translation and activating gene expression programs known globally as the integrated stress response (ISR) [37]. To date, four kinases have been identified to mediate eIF2α phosphorylation: PKR, PERK (protein kinase RNA (PKR)-like ER kinase) [38], GCN2 (general control non-derepressible-2) [39], [40] and HRI (heme-regulated inhibitor) [41], [42]. ER stress–mediated eIF2α phosphorylation is carried out by PERK, which is activated by an excess of unfolded proteins accumulating in the ER lumen [38]. Activated PERK phosphorylates eIF2α, attenuating protein synthesis and triggering the translation of specific molecules such as the transcription factor ATF4, which is necessary to mount part of a particular ISR, known as the unfolded protein response (UPR) [43], [44]. Interestingly DNA viruses, such as HSV, that use the ER as a part of its replication cycle, have been reported to interfere with the ER stress response through different mechanisms, such as the dephosphorylation of eIF2α by the viral phosphatase 1 activator, ICP34.5 [45], [46].

We show here, using SUnSET, a non-radioactive method to monitor protein synthesis [47], that independently of any active viral replication, cytosolic poly I:C detection in mouse embryonic fibroblasts (MEFs) promotes a PKR-dependent mRNA translation arrest and an ISR-like response. During the course of this response, ATF4 and its downstream target, the phosphatase-1 (PP1) cofactor, growth arrest and DNA damage-inducible protein 34 (GADD34, also known as MyD116 and Ppp1r15a) [48], are strongly up-regulated. Importantly, although the translation of most mRNAs is strongly inhibited by poly I:C, that of IFN-ß and Interleukin-6 (IL-6) is considerably increased under these conditions. We further demonstrate that PKR-dependent expression of GADD34 is critically required for the normal translation of IFN-ß and IL-6 mRNAs. We prove the relevance of these observations for antiviral responses using CHIKV as a model: we show that GADD34-deficient MEFs are unable to produce IFN-ß during infection and become permissive to CHIKV. We further show that CHIKV induces 100% lethality in 12-day-old GADD34-deficient mice, whereas WT controls do not succumb to infection. Our observations demonstrate that induction of GADD34 is part of the anti-viral response and imply the existence of distinct and segregated groups of mRNA, which require GADD34 for their efficient translation upon dsRNA-induced eIF2α phosphorylation.

## Results

### Poly I:C induces translational arrest and IFN-ß production

**Figure 1:**
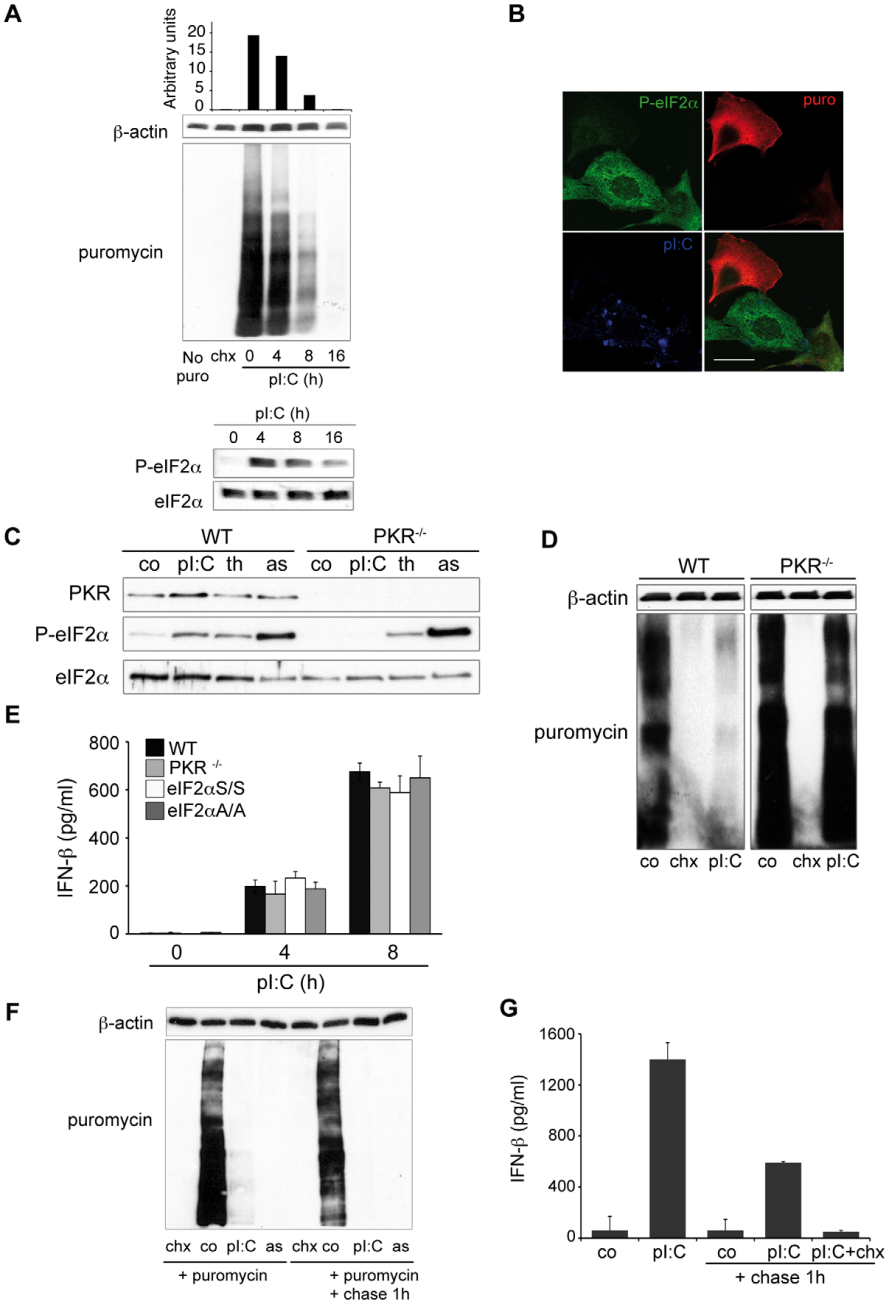
Translation inhibition and IFN-β production are induced by poly I:C in MEFs. **A**) Protein synthesis was monitored in poly I:C(pI:C)-stimulated MEFs using puromycin labelling followed by immunoblot with the anti-puromycin mAb 12D10. Controls are cells not treated with puromycin (No puro) and cells treated with cycloheximide (chx) 5 min prior puromycin incorporation. β-actin immunoblot is shown for equal loading control. Quantification of puromycin signal was quantified with ImageJ software and is represented above the immunoblot. Phosphorylation of eIF2α (P-eIF2α) was assessed in the same MEFs extracts. **B**) Immunofluorescence staining for puromycin, P-eIF2α and dsRNA of MEFs treated with poly I:C for 4 h and labeled with puromycin for 1 h. Scale bar, 10 µm. **C**) WT and PKR^−/−^ MEFs were stimulated for 8 h with poly I:C (pI:C), thapsigargin (th) or arsenite (as). PKR and P-eIF2α were detected by immunoblot. **D**) WT and PKR^−/−^ MEFs were stimulated for 8 h with poly I:C and protein synthesis was monitored like in (A). β-actin immunoblot is shown for equal loading control. **E**) IFN-β levels were measured, by ELISA, in cell culture supernatants of WT, PKR^−/−^, eIF2αA/A and control eIF2αS/S MEFs after 4 and 8 h of poly I:C stimulation. Data are mean ± standard deviation of 3 independent experiments. **F**) Protein synthesis was measured in NIH3T3 cells by puromycin incorporation after 7 h of poly I:C treatment. Where indicated, a chase of 1 h with fresh media was performed prior to puromycin labeling and immunoblotting. Samples with cycloheximide (chx) and arsenite (as) added respectively 5 min and 30 min before the puromycin pulse are shown as controls. **G**) IFN-β was quantified by ELISA in culture supernatants in the conditions described above after 7 h of poly I:C stimulation or 7 h of poly I:C stimulation followed by 1 h with fresh media (chase). Data are mean ± standard deviation of 4 independent experiments.

We monitored protein synthesis in MEFs and NIH-3T3 cells after poly I:C stimulation, using puromycin labeling followed by immunodetection with the anti-puromycin mAb 12D10 [47]. Poly I:C delivery to MEFs and NIH-3T3, rapidly and durably inhibited protein synthesis, concomitant with increased eIF2α phosphorylation (P-eIF2α) (Fig. 1A and Fig. S1A). In MEFs, a strong eIF2α phosphorylation was observed after 4 h of poly I:C treatment, followed by a steady dephosphorylation at later times (Fig. 1A). Protein synthesis arrest was confirmed in individual cells by concomitant imaging of poly I:C delivery, mRNA translation and P-eIF2α (Fig. 1B and Fig. S1B), and with a wide range of dsRNA concentrations (Fig. S1C). Poly I:C-induced eIF2α phosphorylation and subsequent translation arrest were not observed in PKR-deficient MEFs (Fig. 1C and 1D), while eIF2α phosphorylation induced by the UPR-inducing drug thapsigargin (th) (an inhibitor of SERCA ATPases) or arsenite (as) was unchanged in PKR−/− cells (Fig. 1C). PKR is therefore necessary to induce protein synthesis inhibition in response to cytosolic poly I:C.

When levels of IFN-ß were quantified in culture supernatants and compared to total protein synthesis intensity, we found that most of the cytokine production occurred after 4 to 8 h of pIC delivery (Fig. 1E, WT, and S1D), a time at which mRNA translation was already considerably decreased (Fig. 1A and S1E). We measured the amount of cytokine produced in NIH-3T3 cells at a time (7 h) at which translation was already strongly inhibited (Fig. 1G and 1F). To prove that IFN-β production truly occurred during this poly I:C-induced translation arrest, cells exposed for 7 h to poly I:C were washed and old culture supernatants replaced with fresh media for 1 h (with or without CHX), prior translation monitoring (Fig. 1F, right) and IFN-ß dosage (Fig. 1G, right). We observed that close to 30% of the total IFN-ß produced over 8 h of poly I:C stimulation is achieved during this 1 h period, despite a close to undetectable protein synthesis in the dsRNA-treated cells (Fig. 1F). The neo synthetic nature of this IFN was further demonstrated by the absence of the cytokine in CHX-treated cell supernatants. IFN-β production in response to poly I:C is therefore likely to be specifically regulated and occurs to a large extent independently of the globally repressed translational context. As previously observed in MEFs, IFN-ß production in response to poly I:C was independent of PKR (Fig. 1E) [31]. This suggests that although its production occurs during cap-mediated translation inhibition, it does not directly depend on a specialized open reading frame organization, as described for the translation of the mRNAs coding for the UPR transcription factor ATF4 or the SV 26S mRNA upon eIF2α phosphorylation [26], [49]. This hypothesis is also supported by the ability of MEFs expressing the non-phosphorylatable eIF2α Ser^51^ to Ala mutant (eIF2α A/A), to produce normal levels of IFN-ß in response to poly I:C (Fig. 1E), while global translation was not inhibited by poly I:C in these cells (Fig. S2).

### GADD34 expression is induced by cytosolic poly I:C detection

**Figure 2:**
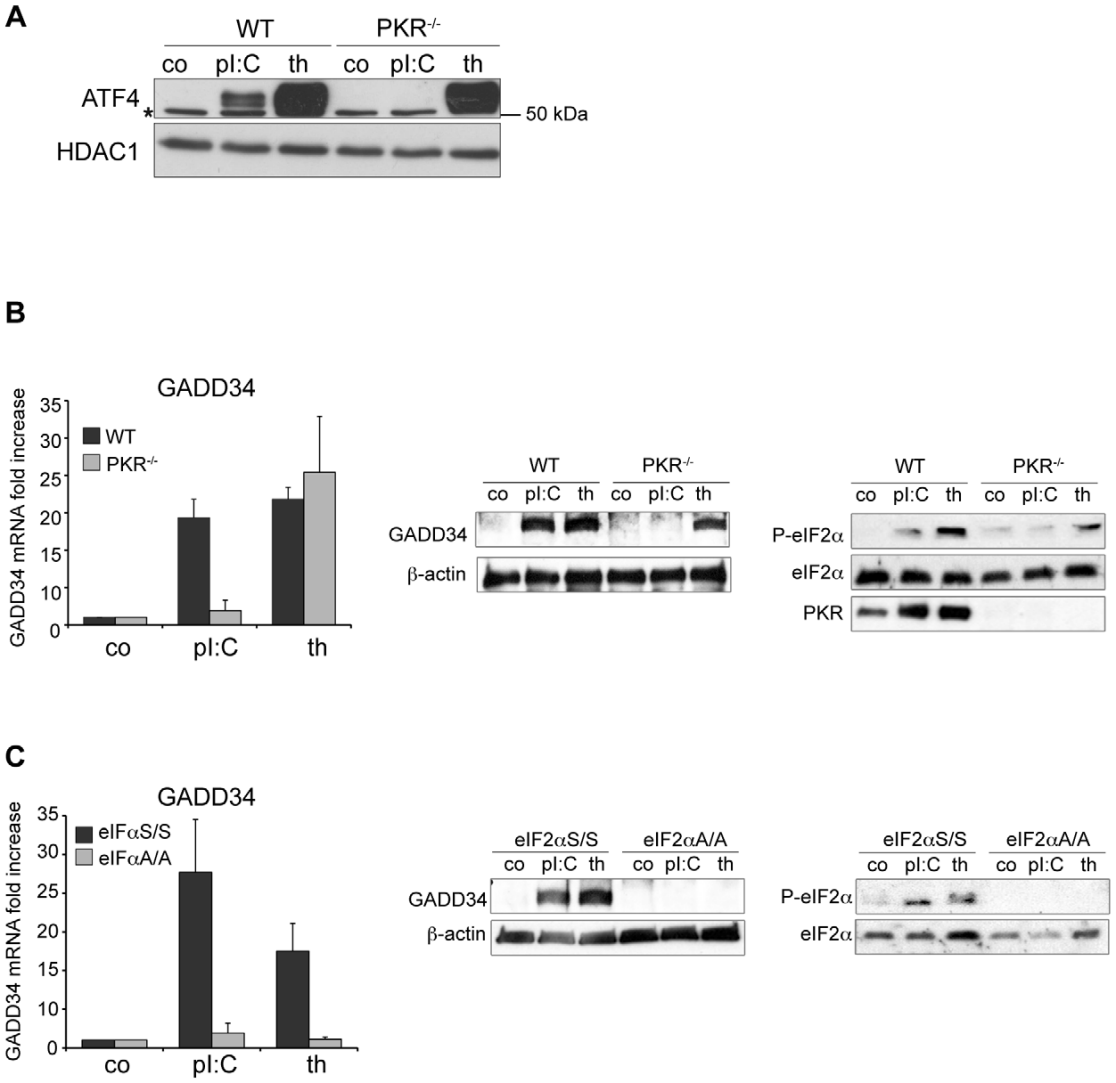
PKR is required for ATF4 and GADD34 expression in response to poly I:C. **A**) WT and PKR^−/−^ MEFs were stimulated for 8 h with poly I:C (pI:C), or the UPR-inducing drug, thapsigargin (th) for 6 h. ATF4 protein expression was detected by immunoblot on nuclear extracts. Nuclear HDAC1 immunoblot is shown for equal loading control. * indicates unspecific band. **B**) GADD34 mRNA levels were quantified by qPCR after 6 h of poly I:C (pI:C) treatment in WT and PKR^−/−^ MEFs. For the same cell extracts, immunoblots of GADD34 (middle panel), PKR and P-eIF2α (right panel) were performed. **C**) The same analysis was performed in eIF2α A/A and control eIF2α S/S MEFs. Treatment with thapsigargin (th), for 6 h was used as control to induce GADD34 and P-eIF2α. eIF2α and β-actin immunoblots are shown for equal loading control. Quantitative PCR data are the mean ± standard deviation of 3 independent experiments.

We went on to investigate the molecular mechanisms promoting this paradoxical IFN-ß synthesis in an otherwise translationally repressed environment. Induction of eIF2α phosphorylation by PERK during ER stress promotes rapid ATF4 synthesis and nuclear translocation, followed by the transcription of many downstream target genes important for the UPR [50]. Similarly, in presence of PKR, nuclear ATF4 levels were found to be up-regulated in MEFs responding to cytosolic poly I:C, albeit less importantly than upon a *bona fide* UPR induced by thapsigargin (Fig. 2A).

One of the key molecules involved in the control of eIF2α phosphorylation is the protein phosphatase 1 co-factor GADD34, which relieves translation repression during ER stress by promoting eIF2α dephosphorylation [50], [51],[52]. GADD34 is a direct downstream transcription target of ATF4 [53]. Expression of GADD34 was quantified by qPCR and immunoblot in WT and PKR^−/−^ MEFs (Fig. 2B). In WT cells GADD34 mRNA expression was clearly up-regulated (20 fold) in response to poly I:C, while GADD34 protein induction was equivalent in poly I:C- and thapsigargin-treated cells. GADD34 mRNA transcription and translation were not observed in PKR^−/−^ cells responding to poly I:C, but occurred normally upon thapsigargin treatment, paragoning eIF2α phosphorylation (Fig. 2B, right).

We next investigated the importance of ATF4 for GADD34 transcription by monitoring the levels of GADD34 mRNA in ATF4-deficient cells. ATF4^−/−^ MEFs displayed higher basal levels of GADD34 mRNA than WT cells. However, in absence of ATF4, MEFs were unable to efficiently induce GADD34 mRNA transcription in response to any of the stimuli tested (Fig. S3). GADD34 mRNA expression was induced only 2 fold in ATF4^−/−^ MEFs exposed to poly I:C, suggesting that its transcription is mostly dependent on ATF4 in this context. We further investigated P-eIF2α requirement for GADD34 expression and found that eIF2α A/A expressing MEFs were incapable of up-regulating GADD34 in response to poly I:C (Fig. 2C). Phosphorylation of eIF2α by PKR in response to cytosolic poly I:C induces therefore a specific integrated stress response (ISR), that allows ATF4 translation, its nuclear translocation and subsequent GADD34 mRNA transcription.

### GADD34 expression is required for global translation recovery in response to thapsigargin but not to poly I:C

**Figure 3:**
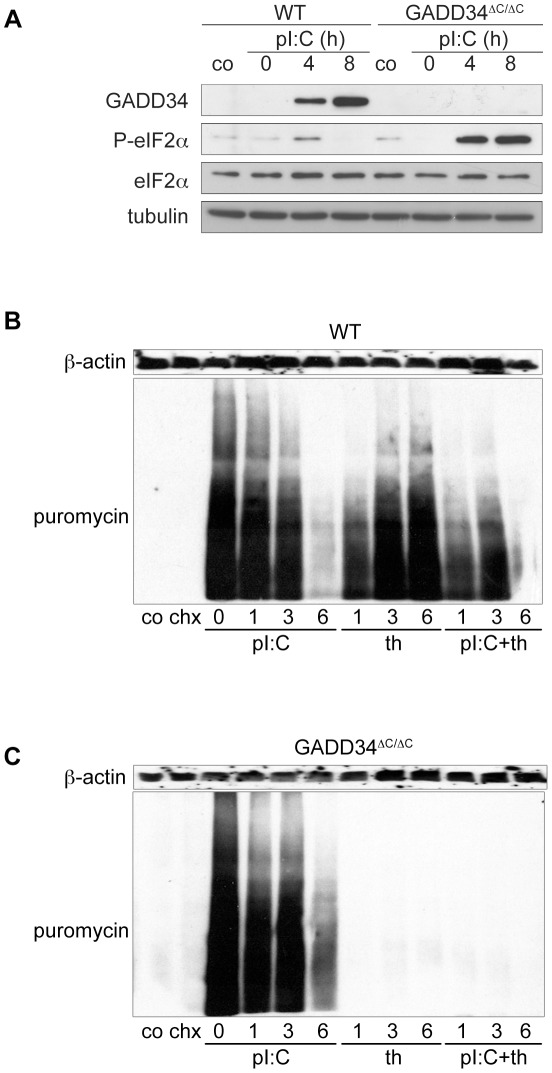
GADD34 mediates eIF2α dephosphorylation but not global translation recovery in response to poly I:C. **A**) After treatment with poly I:C, protein extracts of WT and GADD34^ΔC/ΔC^ MEFs were immunobloted for GADD34 and P-eIF2α. **B**) Protein synthesis was analyzed in WT cells treated for 1 to 6 hours with poly I:C (pI:C) alone or together with thapsigargin (th). Controls are cells not treated with puromycin (co) and cells treated with cycloheximide (chx) 5 min before puromycin incorporation. **C**) Protein synthesis was analyzed in GADD34^ΔC/ΔC^ cells treated for 1 to 6 hours with poly I:C (pI:C) alone or together with thapsigargin (th). Tubulin or β-actin immunoblot are shown for equal loading control. In GADD34^ΔC/ΔC^ cells translation is strongly impacted by thapsigargin, but not poly I:C.

We next evaluated the relevance of GADD34 induction, by treating WT and GADD34^ΔC/ΔC^ fibroblasts with poly I:C or with drugs known to induce ER stress, such as thapsigargin and the N-glycosylation inhibitor tunicamycin [52]. As expected, in WT cells eIF2α phosphorylation was rapidly increased in response to all ISR-inducing stimuli and decreased concomitantly with the expression of GADD34 over time (Fig. 3A and S4) [52]. Consequently eIF2α phosphorylation was greatly increased in GADD34^ΔC/ΔC^ MEFs in all the conditions tested (Fig. 3A and S4A).

In thapsigargin-treated cells, protein synthesis was reduced in the first hour of treatment and rapidly recovered (Fig. 3B) [54]. Poly I:C, however, nearly completely inhibited translation despite active eIF2α dephosphorylation. This was particularly obvious when poly I:C was co-administrated together with thapsigargin. Indeed, poly I:C dominated the response by preventing the translation recovery normally observed after few hours of drug treatment (Fig. 3B). Surprisingly, in absence of functional GADD34, although eIF2α phosphorylation induction by poly I:C was augmented dramatically, no further decrease in protein synthesis was observed upon treatment of GADD34^ΔC/ΔC^ cells with the dsRNA mimic (Fig. 3A and 3C). The functionality of GADD34 in translation restoration was, however, fully demonstrated, when the same cells were treated with thapsigargin, and protein synthesis was completely inhibited by this treatment [52] (Fig. 3C). Thus, cytosolic dsRNA delivery induces a type of protein synthesis inhibition, which requires eIF2α phosphorylation for its initiation, but conversely cannot be reverted by GADD34 induction and subsequent GADD34-dependent eIF2α dephosphorylation.

The potential contribution of the OAS/2-5A/RNAse L system to this P-eIF2α-independent inhibitory process was evaluated by investigating RNA integrity in MEFs exposed to poly I:C. We used capillary electrophoresis to establish precise RNA integrity numbers (RIN) computed from different electrophoretic traces (pre-, 5S-, fast-, inter-, precursor-, post-region, 18S, 28S, marker) and quantify the degradation level of mRNA and rRNA potentially resulting from the activation of this well characterized anti-viral pathway. No major RNA degradation could be observed upon poly I:C delivery (Fig. S5), suggesting that global RNA degradation does not contribute extensively to the long term translation inhibition observed upon poly I:C delivery in our experimental system.

### GADD34 is required for cytokine production induced by poly I:C

**Figure 4:**
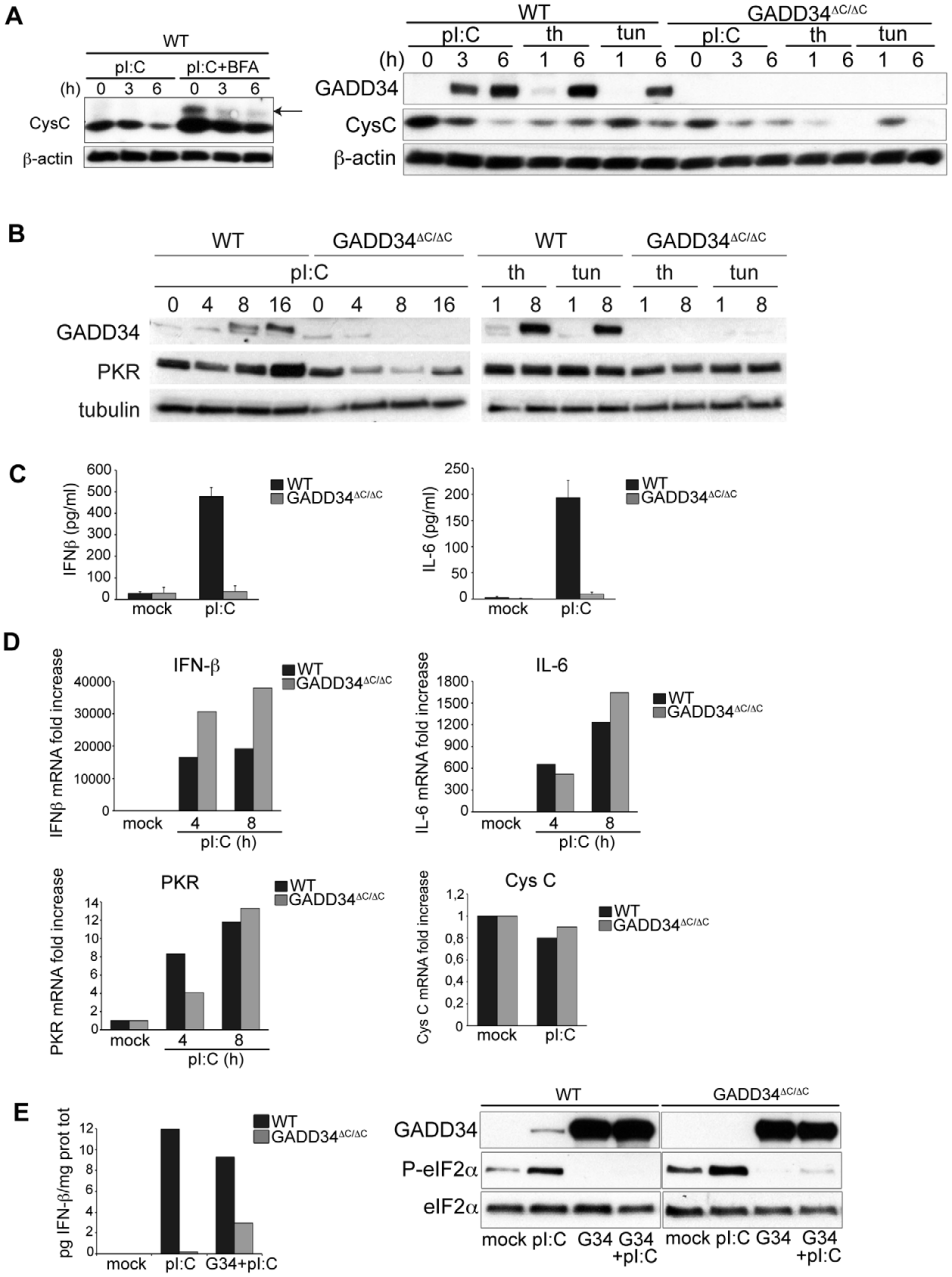
GADD34 is required for cytokine production in poly I:C-stimulated MEFs. **A**) Left Panel, immunoblot for cystatin C after treatment or not with brefeldin A (BFA) in poly I:C-stimulated WT MEFs. Arrow indicates N-glycosylated-Cystatin C. Right panel, WT and GADD34^ΔC/ΔC^ MEFs were treated with poly I:C (pI:C), thapsigargin (th) or tunicamycin (tun) for the indicated times. Levels of GADD34 and Cystatin C (CysC) were examined by immunoblot. β-actin immunoblot is shown as equal loading control. **B**) Immunoblots for GADD34 and PKR in WT and GADD34-inactivated cells treated with poly I:C for the indicated periods of time. The UPR-inducing drugs, Thapsigargin (th) and tunicamycin (tun), were used as controls to induce GADD34. Immunoblot of tubulin is shown as equal loading control. **C**) Amount of IFN-β (left panel) and IL-6 (right panel) in cell culture supernatants of WT and GADD34^ΔC/ΔC^ MEFs after 6 h of poly I:C stimulation. Mock are samples treated with lipofectamine alone. Data are mean ± standard deviation of five (IFN-β) and three (IL-6) independent experiments. **D**) Transcription of IFN-β, IL-6, PKR and Cystatin C was analyzed by qPCR in samples of WT and GADD34^ΔC/ΔC^ MEFs treated with poly I:C (pI:C). Mock represent samples treated with lipofectamine alone. **E**) WT and GADD34^ΔC/ΔC^ MEFs were transfected overnight with an expression plasmid carrying the murine GADD34 (G34) cDNA and then treated with poly I:C for 6 h. IFN-β production was quantified by ELISA, left panel, in cell culture supernatants and plotted as a ratio of IFN-β to total cell proteins to compensate for different cell mortality levels induced by the transfection. In the right panel immunoblots for GADD34 and P-eIF2α in the same experimental conditions are shown. One representative analysis of 3 independent experiments is shown.

We have observed that GADD34 expression counterbalances PKR activation by promoting eIF2α dephosphorylation, however it has little impact on reversing the global translation inhibition initiated by poly I:C. We next monitored the production of specific proteins and cytokines in WT and GADD34^ΔC/ΔC^ MEFs (Fig. 4). Cystatin C, a cysteine protease inhibitor was chosen as a model protein, since its secretion ensures a relative short intracellular residency time so that its intracellular levels directly reflect its synthesis rate [55]. This is confirmed by the N-glycosylated- and total Cystatin C accumulation in cells treated with brefeldin A (Fig. 4A, left panel). Cystatin C levels were found to follow a similar trend to that observed with total translation, being strongly reduced upon poly I:C exposure and not profoundly influenced by GADD34 inactivation (Fig. 4A, right panel). Thapsigargin treatment induced a brief drop in cystatin C levels, prior to some levels of GADD34-dependent recovery. 6 hours of tunicamycin treatment affected more cystatin C accumulation than anticipated (Fig. 4A, right panel), probably due to interference with the N-glycosylation and associated folding of this di-sulfide bridge containing protein [55], thereby promoting its degradation by endoplasmic reticulum-associated protein degradation (ERAD) [56].

We next turned towards PKR, which displayed a pattern of expression completely different from cystatin C (Fig. 4B). As expected from its IFN-inducible transcription, levels of PKR were increased in poly I:C-treated MEFs (Fig. 4B), despite the strong global translation inhibition observed in these cells (Fig. 3). GADD34 inactivation appeared to influence the accumulation of PKR, since the cytoplasmic dsRNA sensor levels were not up-regulated and even decreased in poly I:C-treated GADD34^ΔC/ΔC^ MEFs (Fig. 4B). Control treatment with tunicamycin and thapsigargin did not alter significantly PKR levels (Fig. 4B), suggesting that ER stress did not influence the kinase expression. The absence of PKR up-regulation in the poly I:C-treated GADD34^ΔC/ΔC^ MEFs led us to investigate the capacity of these cells to produce anti-viral and inflammatory cytokines, which normally drive PKR expression through an autocrine loop. We ruled out any interference from the UPR in triggering IFN-ß production in our experimental system, since, as anticipated from PKR expression, tunicamycin and thapsigargin treatments were not sufficient to promote cytokine production in MEFs (Fig. S6) [43], [44].

We therefore investigated IFN-ß and IL-6 production in response to dsRNA in WT, GADD34^ΔC/ΔC^ and CReP^−/−^ MEFs. CReP^−/−^ MEFs were used as a control, since CReP (Ppp1r15b) is a non-inducible co-factor of PP1 and displays some functional redundancy with GADD34 [57]. Although basal levels of eIF2α phosphorylation were higher in CReP^−/−^, PKR expression and translation inhibition upon poly I:C delivery were equivalent in WT and CReP^−/−^ MEFs (Fig. S7A and S7B). Quantification of IFN-ß and IL-6 levels in culture supernatants indicated that, although abundant and comparable amounts of these cytokines were secreted by WT and CReP^−/−^ cells, they were both absent in poly I:C-treated GADD34^ΔC/ΔC^ MEFS (Fig. 4C and S7C).

Quantitative PCR analysis revealed that, IFN-ß, IL-6 and PKR transcripts were potently induced in poly I:C treated GADD34^ΔC/ΔC^ MEFs (Fig. 4D), thus excluding any major transcriptional alterations in these cells, as confirmed by the normal levels of cystatin C mRNA, which remained constant in all conditions studied. Moreover, using confocal immunofluorescence microscopy, we could not detect intracellular IFN-β in poly I:C-stimulated GADD34^ΔC/ΔC^ MEFs, in contrast to WT cells, which abundantly expressed the cytokine, despite the global translation arrest (Fig. S8). Thus, we could attribute the deficit in cytokine secretion of the GADD34^ΔC/ΔC^ MEFs to a profound inability of these cells to synthesize cytokines, rather than to a defect in transcription or general protein secretion.

GADD34 induction by poly I:C is therefore absolutely necessary to maintain the synthesis of specific cytokines and probably several other proteins in an otherwise translationally repressed context. Importantly, GADD34 exerts its rescuing activity only on a selected group of mRNAs including those coding for IFN-ß and IL-6, but not on all ER-translocated proteins, since cystatin C synthesis was strongly inhibited by poly I:C in all conditions tested.

Interestingly, in GADD34^ΔC/ΔC^ MEFs, PKR mRNA strongly accumulated in response to poly I:C (Fig. 4D), despite the absence of detectable IFN-ß production and PKR protein increase (Fig. 4B). This continuous accumulation of PKR mRNA in response to poly I:C suggests the existence of alternative molecular mechanisms, capable of promoting PKR mRNA transcription and stabilization independently of autocrine IFN-β detection. Nevertheless in these conditions PKR expression, like IFN-β, was found to be dependent on the presence of GADD34 for its synthesis (Fig. 4B).

Recent results indicate that PKR participates to the production of IFN-α/ß proteins in response to a subset of RNA viruses including encephalomyocarditis, Theiler's murine encephalomyelitis, and Semliki Forest virus [31]. Even though IFN-α/ß mRNA induction is normal in PKR-deficient cells, a high proportion of mRNA transcripts lack their poly(A) tail [31]. As GADD34 induction by poly I:C was completely PKR-dependent, we wondered whether the phenotypes observed in PKR^−/−^cells and GADD34^ΔC/ΔC^ MEFs could be related. Oligo-dT purified mRNA extracted from cells exposed to poly I:C were therefore analyzed by qPCR. PolyA+ mRNAs coding for IFN-ß and IL-6 were equivalently purified and amplified from WT and GADD34^ΔC/ΔC^ MEFs (Fig. S9). This confirms that albeit the phenotypes of PKR^−/−^ and GADD34^ΔC/ΔC^ cells might be linked, mRNA instability is not the primary cause of the cytokine production defect observed in GADD34^ΔC/ΔC^. Taken together these observations suggest the existence of a specific mRNAs pool, encompassing cardinal immune effectors such as IFN-ß, IL-6, and PKR, which are specifically translated in response to dsRNA sensing and increased levels of P-eIF2α. This mRNAs pool requires GADD34 for their translation during the global protein synthesis shut-down triggered by dsRNA detection.

### GADD34 rescues cytokine production in GADD34^ΔC/ΔC^ MEFs

We verified that GADD34 inactivation, and no other deficiency, was truly responsible for the loss of cytokine production by complementing GADD34^ΔC/ΔC^ MEFs with GADD34 cDNA prior poly I:C delivery. IFN-ß secretion was partially restored in transfected GADD34^ΔC/ΔC^ cells while eIF2α was efficiently dephosphorylated in both WT and GADD34^ΔC/ΔC^ transfected MEFs (Fig. 4E). To further demonstrate that the phosphatase activity of GADD34 controls cytokine production upon dsRNA detection, we treated WT MEFs with guanabenz, a small molecule, which selectively impairs GADD34-dependent eIF2α dephosphorylation [58]. Upon treatment with this compound, a dose dependent inhibition of IFN-ß secretion was observed in poly I:C-treated MEFs, confirming the importance of GADD34 in this process (Fig. S10).

### GADD34 is necessary for IFN production and to control Chikungunya virus infection

Fibroblasts of both human and mouse origin constitute a major target cell of Chikungunya virus (CHIKV) during the acute phase of infection [59]. In adult mice with a totally abrogated type-I IFN signaling, CHIKV-associated disease is particularly severe and correlates with higher viral loads. Importantly, mice with one copy of the IFN-α/ß receptor (IFNAR) gene develop a mild disease, strengthening the implication of type-I IFN signaling in the control of CHIKV replication [59]. Recently, human fibroblasts infection by CHIKV was shown to induce IFN-α/ß mRNA transcription, while preventing mRNA translation and secretion of these antiviral cytokines. CHIKV was found to trigger eIF2α phosphorylation through PKR activation, however this response is not required for the block of host protein synthesis [15].

**Figure 5:**
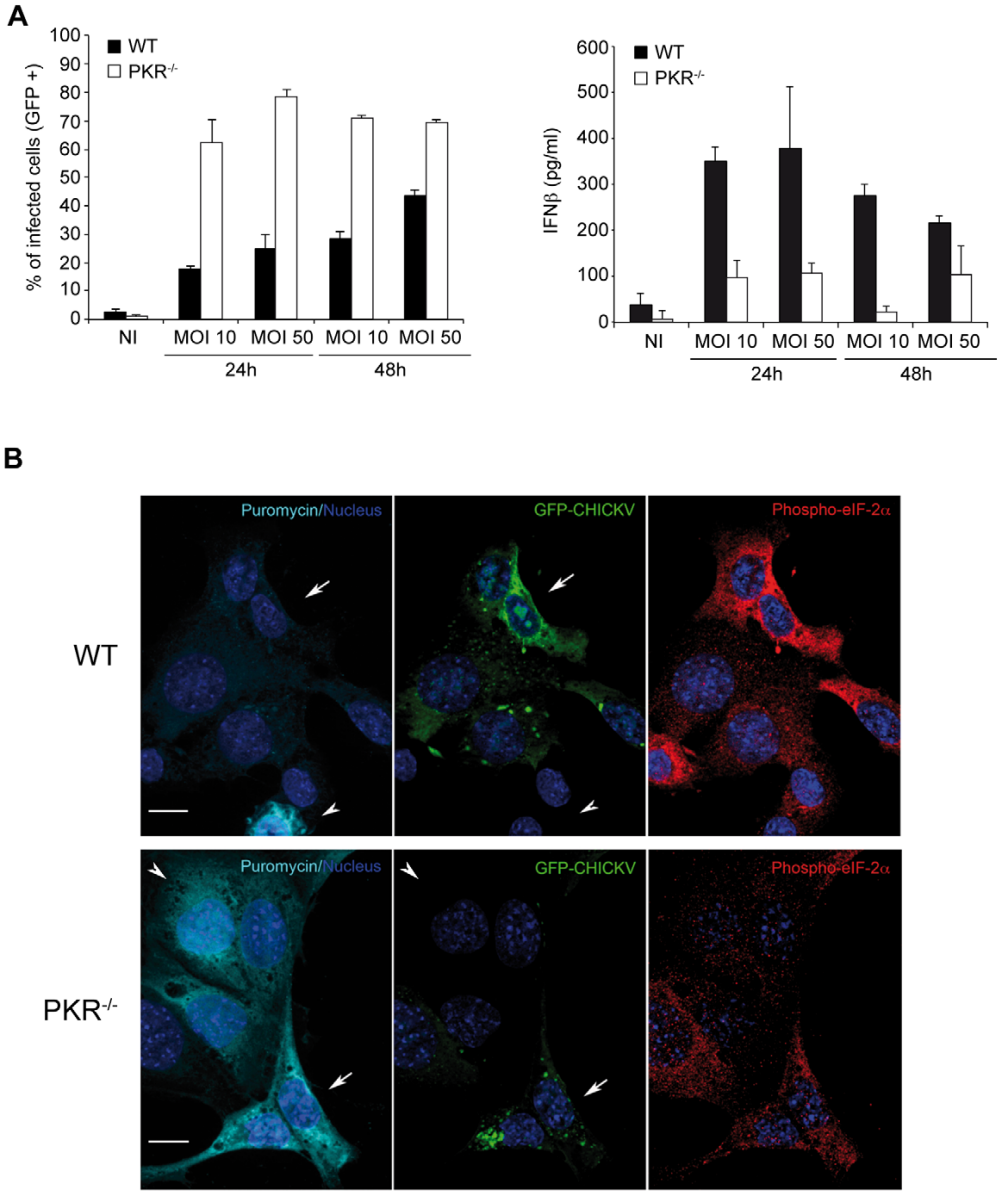
PKR is required to control CHIKV infection and IFN-β production in MEFs. **A**) WT and PKR^−/−^ MEFs were infected with CHIKV-GFP at an MOI of 10 or 50, for 24 h and 48 h. The amount of infected cells was determined by GFP expression, left panel. Interferon β present in the cell culture supernatants was measured by ELISA, right panel. Data represented are mean ± standard deviation from 3 experiments. **B**) WT (top panel) and PKR^−/−^ MEFs (bottom panel) were infected for 24 h with CHIKV-GFP, then labeled with puromycin for 1 h prior fixation. GFP-CHIKV positive (green) were visualized by confocal microscopy after staining with specific antibodies for puromycin (cyan) and phospho-eIF2α (red). Cell Nuclei are stained with Hoechst 33258 (blue). Infection by CHIKV inhibits protein synthesis (visualized by puromycin incorporation) in WT, but not in PKR-deficient cells (arrows). In WT MEFs, eIF2α phosphorylation levels correlate with translation inhibition, although variability is observed among different infected cells, presumably due to GADD34 activity and time of infection. Non-infected WT and PKR^−/−^ cells serve as a reference for normal translation activity and are indicated by an arrowhead. Scale bar 10 µm.

We tested the importance of PKR during CHIKV infection by infecting WT and PKR^−/−^ MEFs with CHIKV-GFP, at a multiplicity of infection (MOI) of 10 and 50. Productive infection was estimated by GFP expression (Fig. 5A, left panel), while culture supernatants were monitored for the presence of IFN-β (5A, right panel). PKR was found to be necessary to control CHIKV infection *in vitro*, since at least 60% of PKR–inactivated cells were infected after 24 of viral exposure, compared to only 15% in the control fibroblasts population. WT MEFs produced efficiently IFN-β, while the hypersensitivity to infection of the PKR^−/−^ MEFs was correlated to a reduced type-I IFN production capacity after infection. Thus, during CHIKV infection, PKR is required for normal IFN production by MEFs. We also monitored protein synthesis in infected WT and PKR^−/−^ fibroblasts using puromycin labeling followed by immunofluorescence confocal microscopy (Fig. 5B). CHIKV-GFP positive PKR^−/−^ MEFs were found to incorporate efficiently puromycin, while in their infected WT counterpart protein synthesis was efficiently inhibited. Thus CHIKV, in this experimental model, induces a PKR-dependent protein synthesis inhibition and is therefore particularly relevant to further confirm our observations on the role of GADD34 in controlling type-I IFN production during response to viral RNAs.

**Figure 6:**
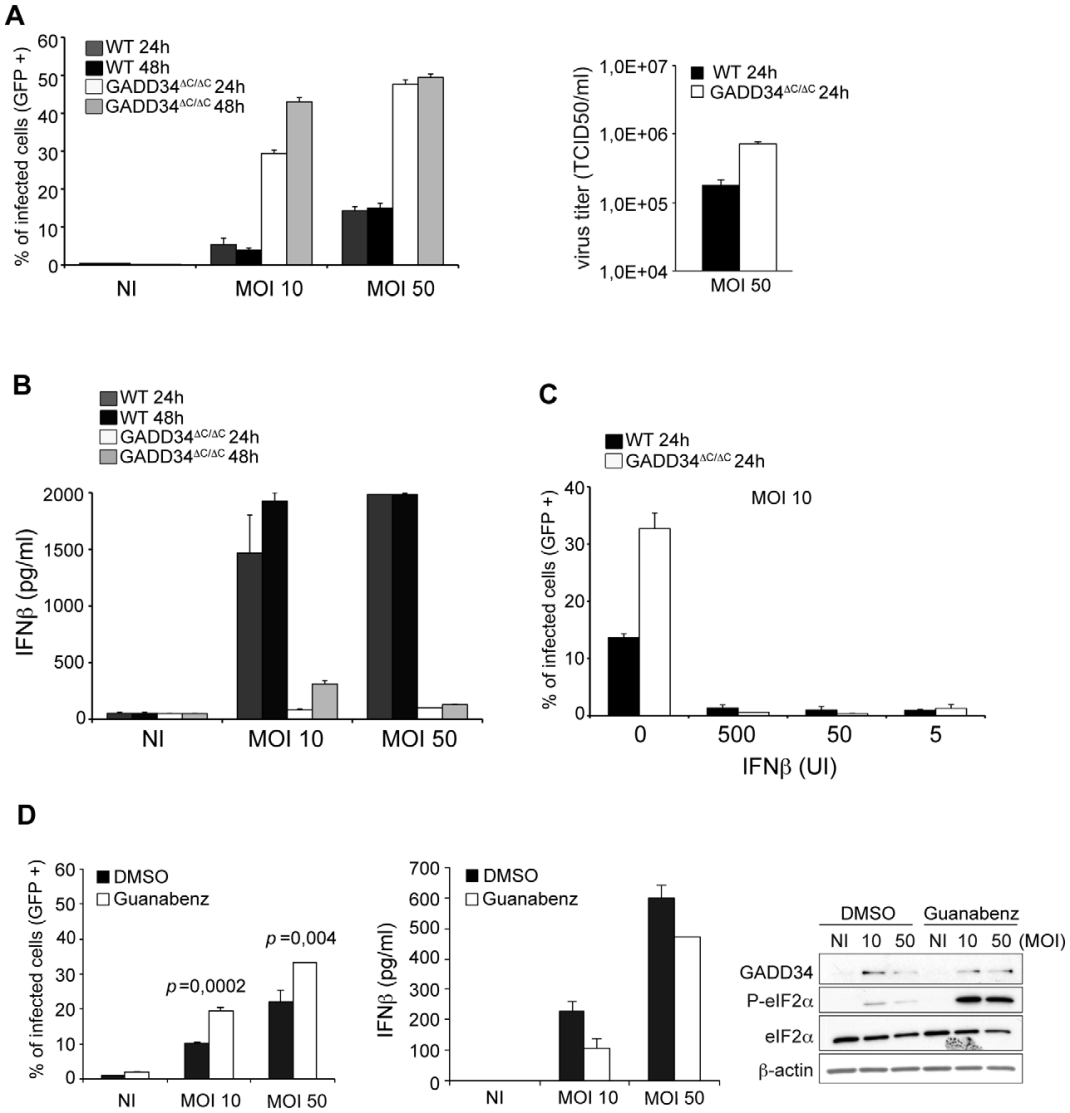
CHIKV infection and IFN-β production are controlled by GADD34 in MEFs. **A**) WT and GADD34^ΔC/ΔC^ cells were infected with CHIKV-GFP for a period of 24 and 48 h. The percentage of infected, GFP positive, cells and viral titers were analyzed. **B**) Levels of IFN-β in cell culture supernatants of WT and GADD34^ΔC/ΔC^ MEFs infected with CHIKV-GFP for 24 h and 48 h. **C**) Murine IFN-β was added 3 h before infection of WT and GADD34^ΔC/ΔC^ MEFs with CHIKV-GFP (10 MOI). Productive infection was estimated by GFP expression 24 h after CHIKV exposure. **D**) Cells were treated with guanabenz 3 h before and during infection (24 h) with CHIKV-GFP, percentage of infected cells (left) and corresponding IFN-β production (middle), are shown. Immunoblot of GADD34 and P-eIF2α are shown on the right. NI stands for non-infected. Percentage of infected cells, viral titers and IFN-β measurements data represent mean ± standard deviation of 3 experiments. *p* values shown in (**D**) were obtained applying a Student's t test.

GADD34^ΔC/ΔC^ MEFs were exposed to CHIKV-GFP (MOI of 10 or 50) for 24 and 48 h. Productive infection was estimated by GFP expression and virus titration (Fig. 6A), and culture supernatants monitored for the presence of type-I IFN (Fig. 6B, left). Only minimal CHIKV infection (15%) could be observed at maximum MOI in WT MEFs (Fig. 6A, left), while robust IFN- β amounts were already produced at the lowest MOI (Fig. 6B). Contrasting with WT cells and regardless of the MOI used, a higher level of viral replication was observed in GADD34^ΔC/ΔC^ MEFs (Fig. 6A). The GADD34-inactivated cells were clearly more sensitive to CHIKV, displaying a 50% infection rate after 24 h of infection (MOI 50) and a log more of virus titer in culture supernatants (Fig. 6A, right). Correlated with their susceptibility to CHIKV infection, IFN-β production was nearly undetectable in GADD34^ΔC/ΔC^ MEFs (Fig. 6B). Such observation confirms the incapacity of GADD34-deficient cells to produce cytokines in response to cytosolic dsRNA, a deficiency likely to facilitate viral replication. This interpretation is further supported by the abrogation of viral replication in both WT and GADD34^ΔC/ΔC^ MEFs briefly treated with IFN-β (Fig. 6C). Thus, GADD34 inactivation does not favor viral replication *per se*, but is critical for type-I IFN production. Interestingly infection levels were found to be higher in PKR−/− than in GADD34 ^ΔC/ΔC^ MEFs, although this difference could be attributed to clonal MEFs variation, it more likely suggests that PKR-dependent translation arrest could be key in preventing early viral replication in this system. In addition, the relatively lower permissivity of GADD34^ΔC/ΔC^ MEFs to infection at high MOI could indicate the existence of GADD34-dependent defense mechanisms, which could be independent from IFN production and eIF2-α dephosphorylation. To strengthen and generalize these observations, we treated a different strain of WT MEFs with guanabenz and examined the consequences for CHIKV infection. Biochemically, GADD34 expression was induced upon CHIKV infection, and guanabenz treatment resulted in a clear increase in eIF2α phosphorylation, demonstrating the importance of GADD34 in limiting this process during infection (Fig. 6D, right). As observed with GADD34^ΔC/ΔC^ cells, pharmacological and RNAi inhibition of GADD34 was found to increase significantly the sensitivity of MEFs to infection, while reducing their IFN-β production (Fig. 6D and S10). Thus, induction of GADD34 and its phosphatase activity during CHIKV infection, *in vitro*, participates to normal type-I IFN production and control of viral dissemination.

Several components of the innate immune response have been shown to impact on the resistance of adult mice and to restrict efficiently CHIKV infection and its consequences *in vivo* [10]. We decided to investigate the importance of GADD34 upon intradermal injections of CHIKV to WT (FVB) and GADD34^ΔC/ΔC^ mice. Neither strain of adult mice was affected by intradermal injections of CHIKV, with little statistically significant differences in the virus titers found in the different organs. Thus, GADD34 deficiency does not annihilate all the sources of type-I IFN in the infected adult animals, a situation exemplified by the capacity of GADD34^ΔC/ΔC^ bone-marrow derived dendritic cells to produce reduced, but measurable IFN-β in response to poly I:C [60]. This also infers that the light impact of GADD34 inactivation on mouse development [61] does not render these animals more sensitive to CHIKV infection.

**Figure 7:**
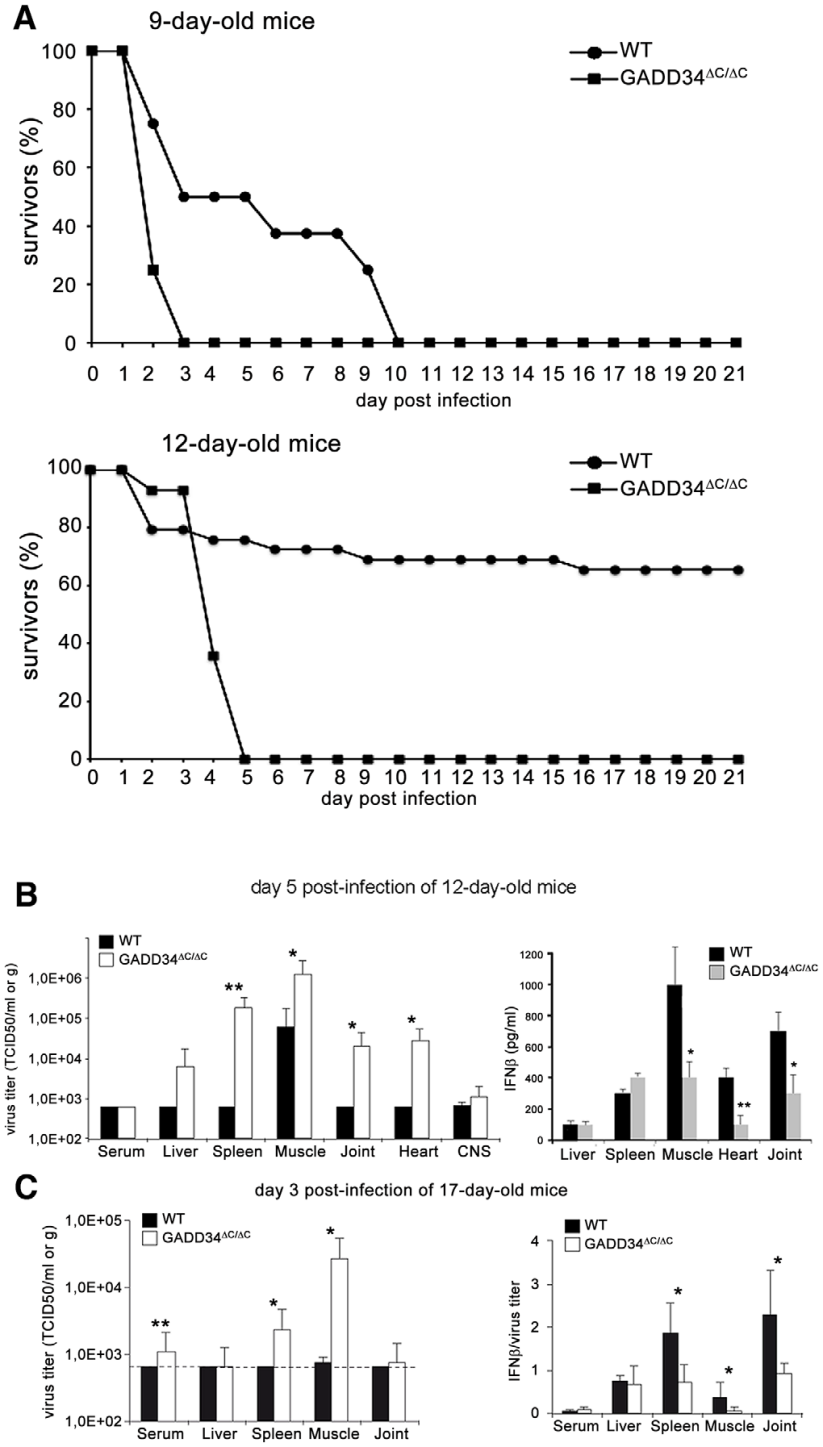
CHIKV infection in mouse neonates. **A**) Kaplan–Meier plots representing the survival of FVB (WT) and GADD34^ΔC/ΔC^ mouse neonates 9-day-old (n = 11 per group) (upper panel) or 12-day-old (n = 14 per group) (lower panel) after intradermal inoculation with 10^6^ PFU of CHIKV and observed for 21 days. **B**) Left panel, viral titers in different tissues and serum of 12-day-old mice inoculated with 10^6^ PFU of CHIKV via the intradermal route. Mice were sacrificed 5 days after infection and the amount of infectious virus in serum and tissues quantified by TCID50 (see methods) (n = 5). In addition of considerably increased levels of viral replication in CHIKV target tissues, GADD34^ΔC/ΔC^ neonates also display signs of heart infection. Right panel, Quantification of IFN-β for the same different tissues, CHIKV-infected target tissues of GADD34^ΔC/ΔC^ mice produced less IFN-β than WT. **C**) 17-day-old mice were infected with 10^6^ PFU of CHIKV via the intradermal route, and sacrificed 72 h later. Quantification of viral titers and IFN-β/viral titers ratio is presented for different tissues. A broken line indicates the detection threshold. In **B** and **C** represented data are arithmetic mean ± standard deviation, n = 5. In **B** and **C** *p* values were calculated using a Student's t test, *p≤0.1, **p≤0.05.

**Figure 8:**
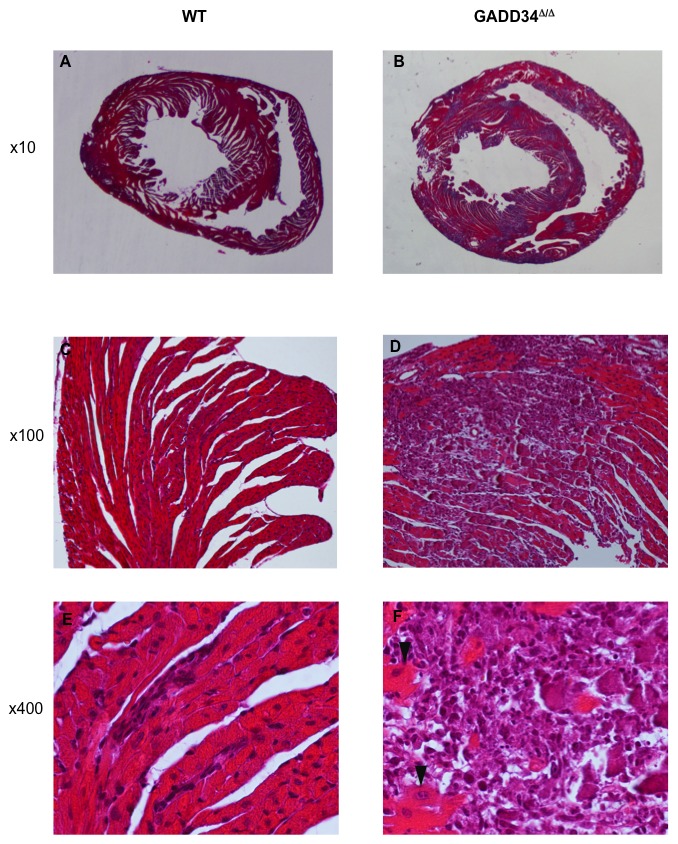
CHIKV infection causes severe myocarditis in mouse neonates. Histological appearance of horizontal sections of the heart through left and right ventricles of 12-day FVB (**A**, **C** and **E**) and GADD34^ΔC/ΔC^ mice at D5 pi (**B**, **D** and **F**). Normal appearance of heart of FVB infected mice, at low magnification (**A**, ×10) with normal cardiomyocytes (**C**, ×100) and exceptional small foci of lymphocytes (**E**, ×400). Numerous foci of necrosis in the heart of GADD34^ΔC/ΔC^ infected mice, at low magnification (**B**, ×10) and extensive through the ventricular wall (**D**, ×100). Higher magnification shows few residual cardiomyocytes (arrow head) and inflammation mainly composed of monocytes as well as extensive deposition of calcium (**F**, ×400). The mice were inoculated with 10^6^ PFU of CHIKV via the intradermal route.

As in Humans, CHIKV pathogenicity is strongly age-dependent in mice, and in less than 12 day-old mouse neonates, CHIKV induces a severe disease accompanied with a high mortality rate [59]. GADD34 function was therefore evaluated in this more sensitive context by injecting intradermally CHIKV to FVB (WT) and GADD34^ΔC/ΔC^ neonatal mice. As previously observed for C57/BL6 mice [59], when CHIKV was inoculated to FVB neonates, a rate of 50% of mortality was observed 3 days after the infection of 9-day-old mice, while 12-day-old pups were found essentially resistant to the virus lethal effect (Fig. 7A). Strongly contrasting with these results, all CHIKV infected GADD34^ΔC/ΔC^ neonates died within 3–5 days post inoculation whatever their age (Fig. 7A). When infection was monitored 5 days post-inoculation of 12-day-old mice at, GADD34^ΔC/ΔC^ pups displayed considerably more elevated CHIKV titers (10–100 folds) in most organs tested, including liver, muscle, spleen and joints, the later being primarily targeted by the virus (Fig. 7B, left). As expected, and in full agreement with the *in vitro* data, infected GADD34^ΔC/ΔC^ tissues showed a considerably reduced IFN-ß production (40–50%) compared to control tissues (Figure 7B, right), while serum levels were reduced by 20% (not shown). Although Infectious virus was poorly detected in the heart of WT animals, elevated titers of virus were observed in the heart of GADD34-deficient pups, matching the limited production of IFN in this organ. We further investigated the possible pathological consequences of cardiac tissue infection by carrying-out comparative histopathology. Hearts of infected GADD34-deficient animals displayed severe cardiomyocytes necrosis with inflammatory infiltrates by monocytes/macrophages and very important calcium deposition (Fig. 8), all being indicative signs of grave necrotic myocarditis. As a consequence, the left ventricles were strongly dilated, being probably the cause of acute cardiac failures and of the important death rate observed in GADD34^ΔC/ΔC^ infected pups. Histology of infected FVB mice hearts was, however, normal with only few inflammatory cells (mainly lymphocytes) observed in the close vicinity of capillaries.

GADD34 expression is therefore necessary to allow normal type-I interferon production during viral infection and to promote the survival of young infected animals. We could circumvent the age-related acquisition of viral resistance in GADD34^ΔC/ΔC^ mice to 17 days, since mice inoculated at that age survived CHIKV inoculation. In these animals, 3 days post-infection, enhanced viral replication was observed in the spleen and muscles, matching the relatively low level of type-IFN production in these tissues (Figure 7C). Functional GADD34 is therefore required to mount a normal innate response against the virus, but in older mice type-I IFN production by non-infected innate cells is probably capable to gradually overcome GADD34-deficiency and limit viral proliferation in vital organs, such as the heart.

## Discussion

Translation inhibition occurs in response to stress, when other cellular activities have to be reassigned or suspended momentarily. We demonstrate here that the activation of PKR by cytosolic dsRNA results in a stress response, leading to ATF4 and GADD34 induction. GADD34 expression has been observed during the infection of cells by different types of viruses [62] or intracellular bacteria such as *Listeria monocytogenes* [63]. Our observations demonstrate that GADD34 expression is a direct consequence of PKR activation and dsRNA sensing. Interestingly, although GADD34 induction by poly I:C promotes eIF2α dephosphorylation, this is not sufficient to prevent global protein synthesis arrest. The uncoupling of efficient eIF2α dephosphorylation from global translation recovery in response to cytosolic poly I:C implies therefore the existence of additional mechanisms inhibiting global translation. The 2-5A/RNAse L pathway does not seem to be sufficiently active in our experimental setting to explain this prolonged protein synthesis inhibition. The cleavage or the inactivation of other translation factors could work in concert with eIF2α to block or affect the efficiency of other individual steps of mRNA translation [64]. For instance, the phosphorylation of translation elongation factor 2 (eEF-2) is also controlled by eIF2α phosphorylation. Thus, Thr56 phosphorylation of eEF-2, which is known to inhibit its translational function by reducing its affinity for ribosomes, could contribute directly to the protein synthesis inhibition induced by PKR activation [65]. Independently of general protein synthesis inhibition, eIF2α dephosphorylation is necessary for the production of specific proteins upon dsRNA-induced translation inhibition. As demonstrated for ATF4, translation of a given mRNA during stress could rely on the structure and organization of its coding sequence, as well as the presence of multiple alternative initiation codons [49]. Surprisingly, functional GADD34 expression was found necessary for the translation of IL-6, IFN-β, and PKR. This observation points to the existence of a distinct group of mRNAs efficiently translated upon dsRNA detection and dependent on GADD34 activity.

GADD34 is extremely short lived and has been shown to accumulate on the ER, when over-expressed [51]. GADD34 could mediate its activity at the ER level and influence differently eIF2α sub-cellular distribution according to the type, localization, and level of activity displayed by the different eIF2α kinases. The strong eIF2α phosphorylation mediated by PKR in response to poly I:C or viral infection and leading to the initiation of translation inhibition, could be circumvented through GADD34 activity solely at the ER level, thereby allowing local cytokine production in absence of other functional protein synthesis. This selectivity for translation of several specific mRNAs among other ER-secreted molecules suggests further that GADD34 dependent mRNAs might display specific features allowing their efficient identification by GADD34 and associated molecules, as well as allowing their translation in presence of minimal levels of active guanine nucleotide exchange factor eIF2B.

GADD34 and PKR are necessary to produce anti-viral cytokines during CHIKV infection, and probably other types of infection. PKR, ATF4 and GADD34 should therefore be considered as an essential module of the innate anti-viral response machinery. The importance of PKR in anti-viral type-I IFN responses has been the object of contradictory reports [30], [31], [66], [67]. Our observations, however, suggest that PKR function should be re-evaluated by integrating the impact of viral detection on cellular translation. In eIF2A/A and PKR^−/−^ cells, cytokine transcription is induced normally following poly I:C detection by DExD/H box RNA helicases, while as expected in these cells, no eIF2α phosphorylation and subsequent host translation inhibition are observed. This lack of translation arrest in the absence of potent eIF2α phosphorylation allows for normal cytokine production during dsRNA detection, with no requirement for an operational GADD34 feedback loop. The importance of PKR and GADD34 for IFN-β and other cytokines production could therefore be directly linked to the efficiency of the cellular translation inhibition induced by RNA viruses, as exemplified here with CHIKV, which in MEFs strongly activates PKR and subsequent protein synthesis inhibition.

GADD34^ΔC/ΔC^ neonates are extremely sensitive to CHIKV infection and display signs of acute myocarditis and ventricles dilatation probably causing recurrent cardiac failures. CHIKV cardiac tropism is not normally observed in WT mouse and inability of heart tissues to produce sufficient type-I IFN in GADD34^ΔC/ΔC^ could allow abnormally high viral replication, myocarditis and dilated cardiomyopathy. Interestingly many cases of myopericarditis induced by CHIKV and leading to dilated cardiomyopathies in infected patients have been reported since the 1970s after the different western Indian Ocean islands and Indian subcontinent disease outbreaks [68], [69]. These particular symptoms and complications might therefore be the consequences of great variation in the tissue-specific type-I IFN levels induced in CHIKV-infected patients, who might display particular polymorphisms in their innate viral sensing pathways increasing their peculiar susceptibility to viral dissemination in the heart.

Importantly, our data reveal a link between pathogen-associated molecular patterns (PAMPs) and the UPR through the activation of the eIF2-α/ATF4 branch [70]. Similarly, several laboratories have reported that TLR stimulation activates the XBP-1 branch of the UPR and that XBP-1 production was needed to promote a sustained production of inflammatory mediators, including IL-6 [71], [72]. Here, we identify GADD34 as a novel functional link between ISR and PAMPs detection in MEFs, required for the production of cytokines including type-I IFN. It will now be important to explore the therapeutic potential of targeting GADD34 to reduce cytokines overproduction during inflammatory conditions.

## Materials and Methods

### Ethics statement

This study was carried out in strict accordance with the recommendations in the Guide for the Care and Use of Laboratory Animals the French Ministry of Agriculture and of the European Union. The protocol was approved by the Committee on the Ethics of Animal Experiments of the Institut Pasteur and Région PACA (Autorisation # 13.116 issued by DDSV/Préfecture des Bouches du Rhône, Marseille, France) and were performed in compliance with the NIH Animal Welfare Insurance #A5476-01 issued on 02/07/2007. All experiments were performed under isoflurane anesthesia (Forene, Abbott Laboratories Ltd, United-Kingdom), and all efforts were made to minimize suffering. Animals were housed in the Institut Pasteur and CIML animal facilities accredited by the French Ministry of Agriculture to perform experiments on live mice.

### Cells

Matched wild-type (129 SvEv), and PKR^−/−^ MEFs (Yang et al., 1995) were a gift from Caetano Reis e Sousa (Cancer Research UK, London); primary eIF2α S/S and eIF2α A/A MEFs were a gift from Randal J. Kaufman (Department of Biological Chemistry, University of Michigan Medical Center, USA); Matched wild-type (129 SvEv), ATF4^−/−^, GADD34^ΔC/ΔC^ and CReP^−/−^ MEFs were a gift from David Ron (Skirball Institute of Biomolecular Medicine, New York). All MEFs were cultured in DMEM, 10% FCS (HyClone, Perbio), 100 units/ml penicillin, 100 µg/ml streptomycin, 2 mM glutamine, 1× MEM non-essential amino acids and 50 µM 2-mercaptoethanol. NIH3T3 cells were cultured in RPMI 1640 (Gibco) supplemented with 10% FCS (HyClone, PERBIO), 100 units/ml penicillin and 100 µg/ml streptomycin. All cells were cultured at 37°C and 5% CO_2_. MEFs and NIH3T3 were treated for the indicated time with 10 µg/ml poly I:C (InvivoGen) in combination with lipofectamine 2000 (Invitrogen). Thapsigargin, tunicamycin, sodium arsenite, and guanabenz (all from SIGMA) were used at 200 nM, 2 µg/ml, 0.5 mM, and 10 µM respectively. The plasmid GADD34 (FLAG epitope tagged at N-terminus, CMV2-based mammalian expression) was a kind gift from David Ron (Institute of Metabolic Sciences, University of Cambridge, UK).

### Translation intensity measurement

Puromycin labelling for measuring the intensity of translation was performed as previously described [47]. For immunoblots, 10 µg/ml puromycin (Sigma, min 98% TLC, cell culture tested, P8833, diluted in PBS) was added in the culture medium and the cells were incubated for 10 min at 37°C and 5% CO_2_. Where indicated, 25 µM cycloheximide (Sigma) was added 5 min before puromycin. Cells were then harvested, centrifuged at 4°C and washed with cold PBS prior to cell lysis and immunoblotting with the 12D10 antibody.

### Immunoblotting

Cells were lysed in 1% Triton X-100, 50 mM Hepes, 10 mM NaCl, 2.5 mM MgCl_2_, 2 mM EDTA, 10% glycerol, supplemented with Complete Mini Protease Inhibitor Cocktail Tablets (Roche). Protein quantification was performed using the BCA Protein Assay (Pierce). 25–50 µg of Triton X-100-soluble material was loaded on 2%–12% gradient or 8% SDS-PAGE before immunoblotting and chemiluminescence detection (SuperSignal West Pico Chemiluminescent Substrate, Pierce). Nuclear extraction was performed using the Nuclear Complex Co-IP kit (Active Motif). Rabbit polyclonal antibodies recognizing ATF4 (CREB-2, C-20), GADD34 (C-19), Lamin A (H-102) and eIF2-α (FL-315) were from Santa Cruz Biotechnology, as well as mouse monoclonal anti-PKR (B-10). GADD34/PPP1R15A (Catalog No. 10449-1-AP) rabbit polyclonal antibody was purchased from PROTEINTECH.

Rabbit polyclonal anti-eIF2α[pS^52^] and Cystatin C were from Invitrogen and Upstate Biotechnology, respectively. Mouse monoclonal antibodies for β-actin and HDAC1 (10E2) were purchased from Sigma and Cell Signaling Technologies. Secondary antibodies were from Jackson ImmunoResearch Laboratories.

### Immunofluorescence

MEFs and NIH3T3 were grown on coverslips overnight and stimulated for the indicated time with poly I:C complexed with Lipofectamine 2000. Cells were fixed with 3% paraformaldehyde in PBS for 10 min at room temperature, permeabilized with 0,5% saponin in 5% FCS PBS with 100 mM glycine, for 15 min at room temperature and stained for 1 h with indicated primary antibodies. Anti-P-eIF2α was from BioSource; anti-dsRNA (clone K1) from English & Scientific Consulting Bt.; anti-IFN-β-FITC-conjugated from PBL Interferon Source; anti-puromycin (clone 2G11, mouse IgG1) has been previously described [47]. Alexa-conjugated secondary antibodies (30 min staining) were from Molecular Probes (Invitrogen). Coverslips were mounted on a slide and images taken with a laser-scanning confocal microscope (LSM 510; Carl Zeiss MicroImaging) using a 63× objective and accompanying imaging software. When PKR WT and PKR^−/−^ were infected with CHIKV, protocol was performed as follows: cells were fixed with 4% paraformaldehyde in PBS for 20 min, then permeabilized for 30 min in 0.1% Triton 100X (Sigma) and blocked in 10% of normal goat serum (Vector Laboratories). Cells were stained with a mouse monoclonal antibody directed against CHIKV capsid coupled to Alexa-488 and a mouse antibody against puromycin coupled to Alexa-555 and a rabbit antibody anti-eIF2α[pS^52^] (Invitrogen) and a Cyanin-3 secondary antibody, and finally counterstained with Hoechst (Vector Lab). Cells were observed with an AxioObserver microscope (Zeiss). Pictures and Z-stacks were obtained using the AxioVision 4.5 software.

### ELISA

IFN-β and IL-6 quantification in culture supernatant was performed using the Mouse Interferon Beta ELISA kit (PBL InterferonSource) and Mouse Interleukin-6 ELISA kit (eBioscience) respectively, according to manufacturer instructions.

### Quantitative PCR

Total RNA was isolated from cells using the RNeasy miniprep kit (QIAGEN) combined with a DNA digestion step (RNase-free DNase set, QIAGEN). cDNA was synthesized using the Superscript II reverse transcriptase (Invitrogen) and random hexamer primers. Quantitative PCR amplification was carried out using complete SYBR Green PCR master mix (Applied Biosystems) and 200 nM of each specific primer. 5 µl of cDNA template was added to 20 µl of PCR mix, and the amplification was tracked via SYBR Green incorporation by an Applied Biosystems thermal cycler. cDNA concentration in each sample were normalized by using HPRT. A nontemplate control was also routinely performed. The primers used for gene amplification (designed with Primer3 software) were the following: GADD34 (S 5′-GACCCCTCCAACTCTCCTTC-3′, AS 5′-CTTCCTCAGCCTCAGCATTC-3′); HPRT (S 5′-AGGCCAGACTTTGTTGGATTT -3′, AS 5′-GGCTTTGTATTTGGCTTTTCC -3′); IFN-β (S 5′-CCCTATGGAGATGACGGAGA-3′, AS 5′-ACCCAGTGCTGGAGAAATTG-3′); IL-6 (S 5′-CATGTTCTCTGGGAAATCGTG-3′, AS 5′-TCCAGTTTGGTAGCATCCATC-3′); PKR (S 5′-CCGGTGCCTCTTTATTCAAA -3′, AS 5′-ACTCCGGTCACGATTTGTTC-3′); Cystatin C (S 5′-GAGTACAACAAGGGCAGCAAC-3′, AS 5′-TCAAATTTGTCTGGGACTTGG-3′). ATF4 (5′-GGACAGATTGGATGTTGGAGA-3′, AS 5′-AGAGGGGCAAAAAGATCACAT3-′).

mRNA isolation from total RNA was performed with oligodT columns (Genelute mRNA miniprep kit (Sigma). Data were analyzed using the 7500 Fast System Appled Biosystems software.

### RNA integrity measurement

RNA integrity upon poly I:C stimulation was measured by capillary electrophoresis using the the Agilent RNA 6000 Pico Chip kit (Agilent Technologies) in an Agilent 2100 Bioanalyser, according to manufacturer instructions.

### MEFs infection with CHIKV

GADD34^ΔC/ΔC^ and the corresponding WT control MEFs were infected at a multiplicity of infection (MOI) of 10 or 50 with CHIKV-GFP generated using a full-length infectious cDNA clone provided by S. Higgs [71]. By 24 h and 48 h post infection, 30 000 cells were analyzed in triplicate by FACS for expression of GFP. At the same time-points, culture supernatants were collected and IFN-β protein assessed by ELISA. In experiments with exogenous IFN-β, cells were treated with mouse IFN-β (PBL InterferonSource) for 3 h before infection with CHIKV-GFP. When guanabenz was used to specifically inhibit GADD34, MEFs cells were treated for 2 h with 10 µM of Guanabenz or DMSO and then infected in the same medium. Three hours post infection the inoculum was removed and fresh medium with Guanabenz or DMSO was added and maintained all along the experiment. RNAi for GADD34 was performed as described in [60].

### CHIKV infection in mice

FVB WT mice were obtained from Charles River Laboratories (France). GADD34^ΔC/ΔC^ FVB mice were obtained from L. Wrabetz (Milan). Mice were anesthetized and inoculated via the intradermal route with 10^6^ PFU of CHIKV-21 isolate [72]. Viral titers in tissues and serum were determined as described before [59], and expressed as tissue cytopathic infectious dose 50 (TCID50)/g or TCID50/ml, respectively. Organs including heart, liver, skeletal muscles and spleen were collected for histopathological procedures. organs were then fixed in 4% paraformaldehyde solution, paraffin-embedded, sectioned coronally in 5–10 µm thickness and stained with hematoxylin-eosin.

## Supporting Information

Figure S1 **Poly I:C stimulation induces protein translation inhibition and IFN-β production in NIH3T3 cells.** **A**) Protein synthesis was quantified in poly I:C-stimulated NIH3T3 using puromycin labeling followed by immunoblot with anti-puromycin mAb 12D10. Protein synthesis was strongly reduced upon poly I:C stimulation. Immunoblot for phosphorylated (P-eIF2α) and total eIF2α were performed on the same NIH3T3 extract. Cycloheximide (chx) was added 5 min before puromycin incorporation. β-actin immunoblot is shown for equal loading control. **B**) Puromycin integration was analysed by immunofluorescence in NIH3T3 cells treated for 4 h with poly I:C and labeled with puromycin in the last 10 min. Fluorescence intensity profiles were generated with LSM 510 Carl Zeiss MicroImaging software. Upper profile refers to the red line in the upper cell (no poly I:C); lower profile refers to the red line in the lower cells (poly I:C-transfected). The puromycin intensity (green) decreases in the presence of poly I:C (red). Data shown in (**A**) and (**B**) are representative of three independent experiments with similar results. Scale bar, 10 µm. **C**) Protein synthesis quantification of WT MEFs treated for 8 h with different doses of poly I:C. One of two independent experiments with similar results is shown. **D**) IFN-β was quantified in cell culture supernatants of NIH3T3 cells after treatment with poly I:C. **E**) Protein translation was monitored in the same experimental conditions by immunoblot. One of two independent experiments with similar results is shown in (**D**) and (**E**).
(TIF)

Figure S2 **Protein translation in cells with non-phosphorylatable eIF2α.** Protein synthesis was quantified in MEFs with non-phosphorylatable eIF2α, eIF2αA/A and the corresponding control cells, eIF2αS/S. After poly I:C or thapsigargin treatment, puromycin labeling followed by immunoblot, was performed. Puromycin labeling was quantified with ImageJ software and protein translation was depicted as percentage of steady state. Cycloheximide (chx) was added 5 min before puromycin incorporation. Tubulin immunoblot is shown for equal loading control. One of two independent experiments with similar results is shown.
(TIF)

Figure S3 **GADD34 mRNA induction in ATF4-deficient MEFs stimulated with cytosolic poly I:C.** The levels of GADD34 transcript were determined by qPCR in WT and ATF4^−/−^ cells after 8 h of poly I:C stimulation. Treatment with tunicamycin and thapsigargin were used as positive controls for GADD34 induction. Results are displayed according to both WT internal reference (left) and ATF4^−/−^ internal reference (right).
(TIF)

Figure S4 **GADD34 mediates eIF2α dephosphorylation in MEFs stimulated with poly I:C.** **A**) Wild-type and GADD34^ΔC/ΔC^ MEFs were treated for the indicated times with poly I:C (pI:C), tunicamycin (tun) or thapsigargin (th) and eIF2α phosphorylation was monitored by immunoblot. **B**) GADD34 expression was analyzed by immunoblot in samples treated for 1 or 6 hours with poly I:C alone or together with tunicamycin (tun) or thapsigargin (th). Data shown in (**A**) and (**B**) are representative of three independent experiments with similar results.
(TIF)

Figure S5 **RNA integrity upon poly I:C exposure.** WT MEFs were treated with poly I:C for the indicated times and RNA integrity evaluated by capillary electrophoresis (Agilent RNA 6000). RNA Integrity Numbers (RIN) between 8.2 and 9.2 were obtained, indicating a high level of RNA integrity. Data shown are representative of three independent experiments with similar results.
(TIF)

Figure S6 **UPR-inducing drugs do not elicit IFN-β production.** Cell culture supernatants of murine embryonic fibroblasts were tested for the presence of IFN-β, after treatment with poly I:C (8 h), tunicamycin and thapsigargin (6 h). The results shown are representative of 4 experiments.
(TIF)

Figure S7 **Deletion of the constitutively-expressed PP1 co-factor, CReP, does not impact protein translation and IFN-β production in MEFs.** **A**) WT and CReP^−/−^ MEFs were treated with poly I:C (pI:C) for the indicated times and the levels of P-eIF2α and PKR were analyzed by immunoblot. Although basal levels of P-eIF2α were higher in CReP^−/−^ MEFs, increase of phosphorylation upon poly I:C exposure was similar to the WT. PKR expression upon poly I:C treatment was equivalent in CReP^−/−^ and WT MEFs. **B**) Protein synthesis was quantified using puromycin labeling followed by immunoblot with the anti-puromycin mAb 12D10. Where indicated, cells were treated with cycloheximide (chx) 5 min before puromycin incorporation. No major differences were found between WT and CReP^−/−^ cells at the level of translation inhibition following poly I:C exposure. **C**) IFN-β quantification in cell culture supernatants after 8 h of poly I:C (pI:C) treatment. Data shown in this figure are representative of two independent experiments with similar results.
(TIF)

Figure S8 **GADD34 is necessary for IFN-β production in response to poly I:C stimulation.** WT and GADD34^ΔC/ΔC^ MEFs were treated with poly I:C for 8 h and labeled with puromycin for the last 10 min. Immunofluorescence staining for intracellular IFN-β, puromycin (red) and dsRNA (poly I:C, blue) was performed and samples were imaged by confocal microscopy. Scale bar, 10 µm. Data shown are representative of three independent experiments with similar results.
(TIF)

Figure S9 **IFN-β and IL-6 polyA^+^ mRNAs are induced equally in WT and GADD34^ΔC/ΔC^ MEFs in response to dsRNA.** WT and GADD34^ΔC/ΔC^ MEFs were treated for 6 h with poly I:C, total RNA extracted and poly A^+^ mRNAs purified on an oligo-dT column. Quantitative PCR was performed after reverse transcription. Data shown are representative of two independent experiments with similar results.
(TIF)

Figure S10 **Specific inhibition of GADD34 with guanabenz or by RNAi decreases IFN-β production.** **A**) WT MEFs were treated with different doses of guanabenz (or DMSO as control) during 2 hours before being stimulated with poly I:C for 8 hours in the presence or absence of guanabenz. IFN-β levels were monitored in cell culture supernatants after the treatments. Guanabenz decreased IFN-β levels in a dose-dependent fashion. Data shown is representative of three independent experiments with similar results. **B**) MEFs treated with con and GADD34 siRNAs were infected with CHIKV-GFP for a period of 24 h. The percentage of infected GFP positive cells and resulting IFN-ß production were analyzed.
(TIF)
