# Supplementary figures and images for: Induction of GADD34 Is Necessary for dsRNA-Dependent Interferon-β Production and Participates in the Control of Chikungunya Virus Infection
Source: PLoS Pathog. 2012 May 17;8(5):e1002708. doi: 10.1371/journal.ppat.1002708 (PMC3355096; doi:10.1371/journal.ppat.1002708)

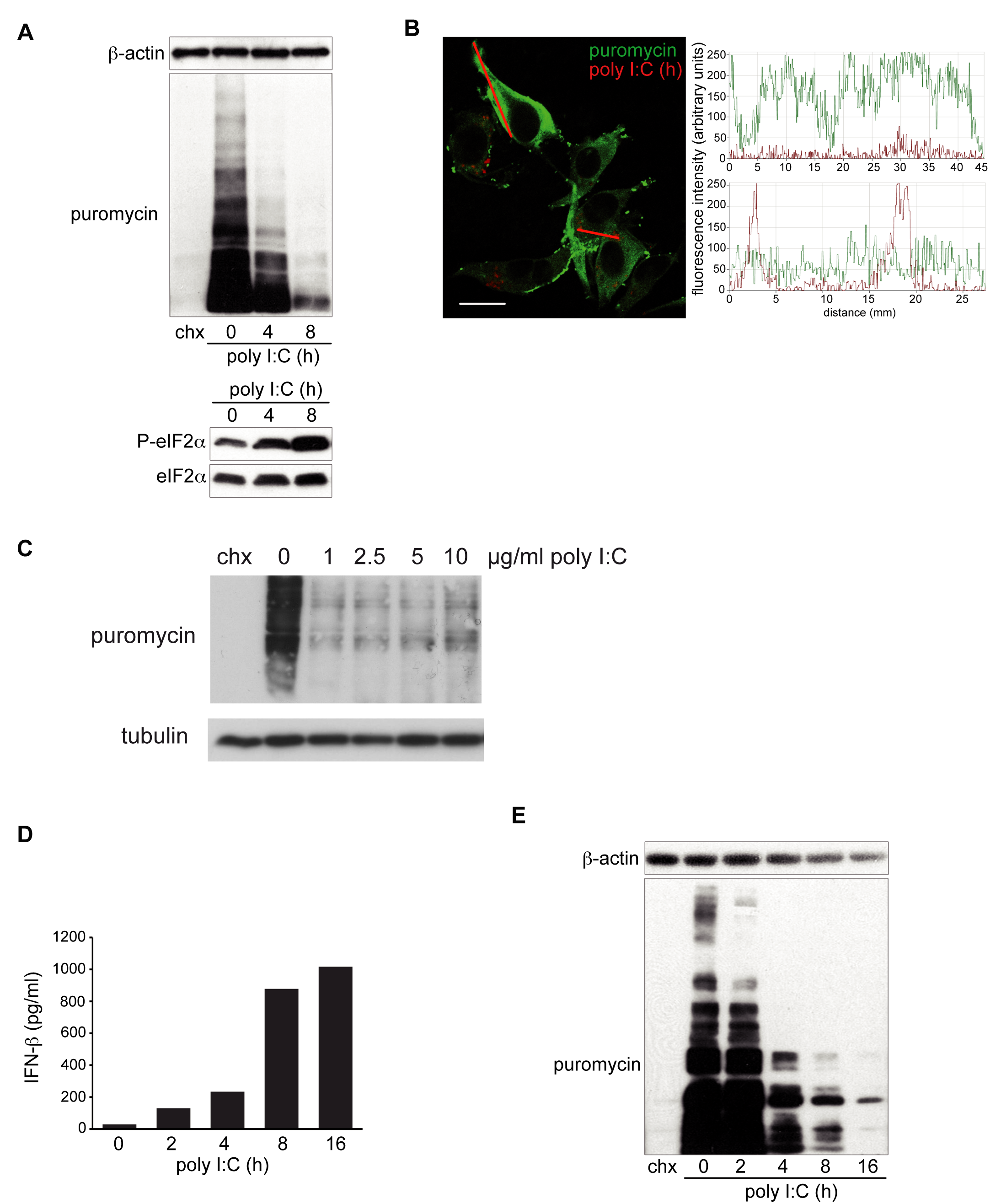

Supplement: Figure S1 — Poly I:C stimulation induces protein translation inhibition and IFN-β production in NIH3T3 cells. A) Protein synthesis was quantified in poly I:C-stimulated NIH3T3 using puromycin labeling followed by immunoblot with anti-puromycin mAb 12D10. Protein synthesis was strongly reduced upon poly I:C stimulation. Immunoblot for phosphorylated (P-eIF2α) and total eIF2α were performed on the same NIH3T3 extract. Cycloheximide (chx) was added 5 min before puromycin incorporation. β-actin immunoblot is shown for equal loading control. B) Puromycin integration was analysed by immunofluorescence in NIH3T3 cells treated for 4 h with poly I:C and labeled with puromycin in the last 10 min. Fluorescence intensity profiles were generated with LSM 510 Carl Zeiss MicroImaging software. Upper profile refers to the red line in the upper cell (no poly I:C); lower profile refers to the red line in the lower cells (poly I:C-transfected). The puromycin intensity (green) decreases in the presence of poly I:C (red). Data shown in (A) and (B) are representative of three independent experiments with similar results. Scale bar, 10 µm. C) Protein synthesis quantification of WT MEFs treated for 8 h with different doses of poly I:C. One of two independent experiments with similar results is shown. D) IFN-β was quantified in cell culture supernatants of NIH3T3 cells after treatment with poly I:C. E) Protein translation was monitored in the same experimental conditions by immunoblot. One of two independent experiments with similar results is shown in (D) and (E). (TIF) [file ppat.1002708.s001.tif]

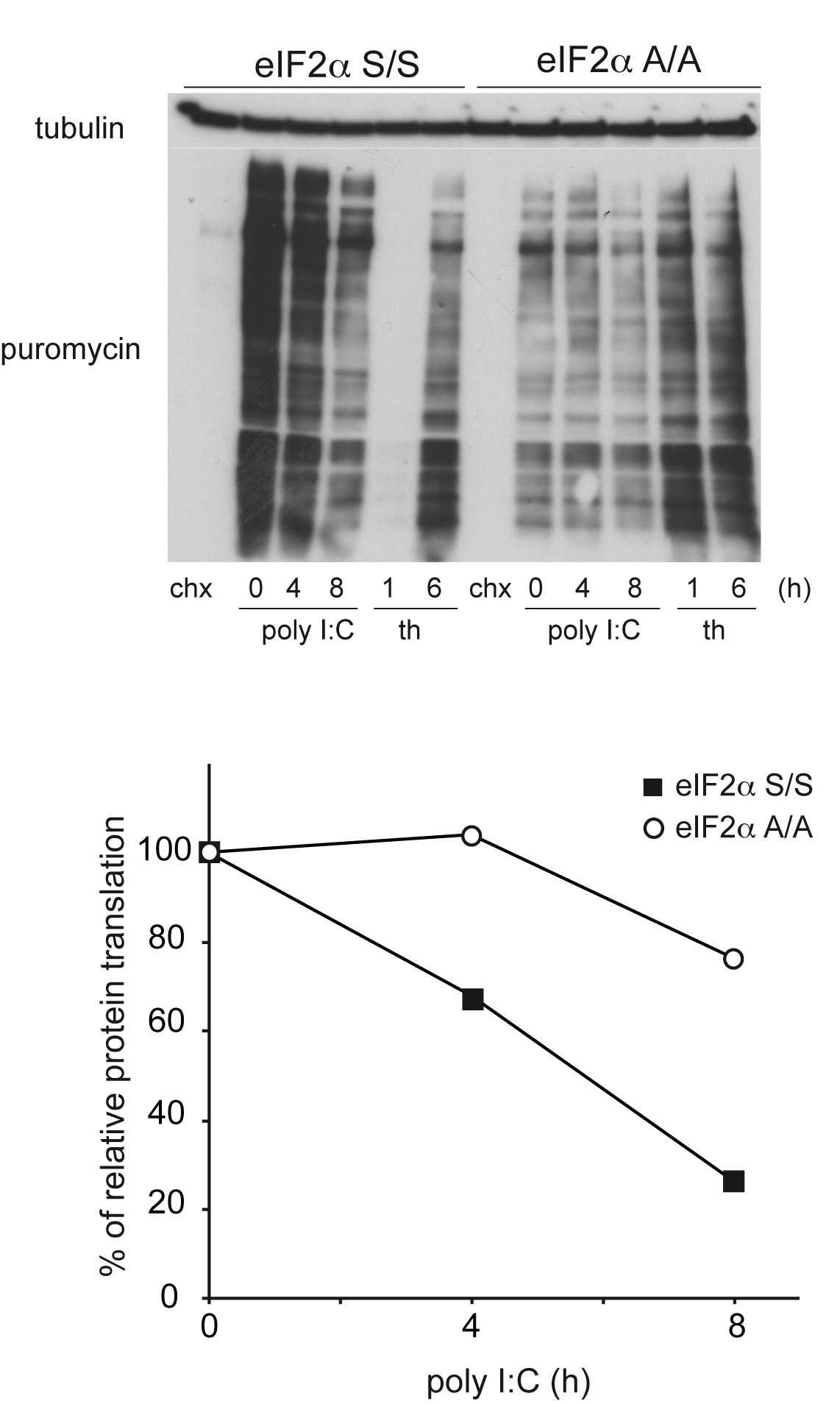

Supplement: Figure S2 — Protein translation in cells with non-phosphorylatable eIF2α. Protein synthesis was quantified in MEFs with non-phosphorylatable eIF2α, eIF2αA/A and the corresponding control cells, eIF2αS/S. After poly I:C or thapsigargin treatment, puromycin labeling followed by immunoblot, was performed. Puromycin labeling was quantified with ImageJ software and protein translation was depicted as percentage of steady state. Cycloheximide (chx) was added 5 min before puromycin incorporation. Tubulin immunoblot is shown for equal loading control. One of two independent experiments with similar results is shown. (TIF) [file ppat.1002708.s002.tif]

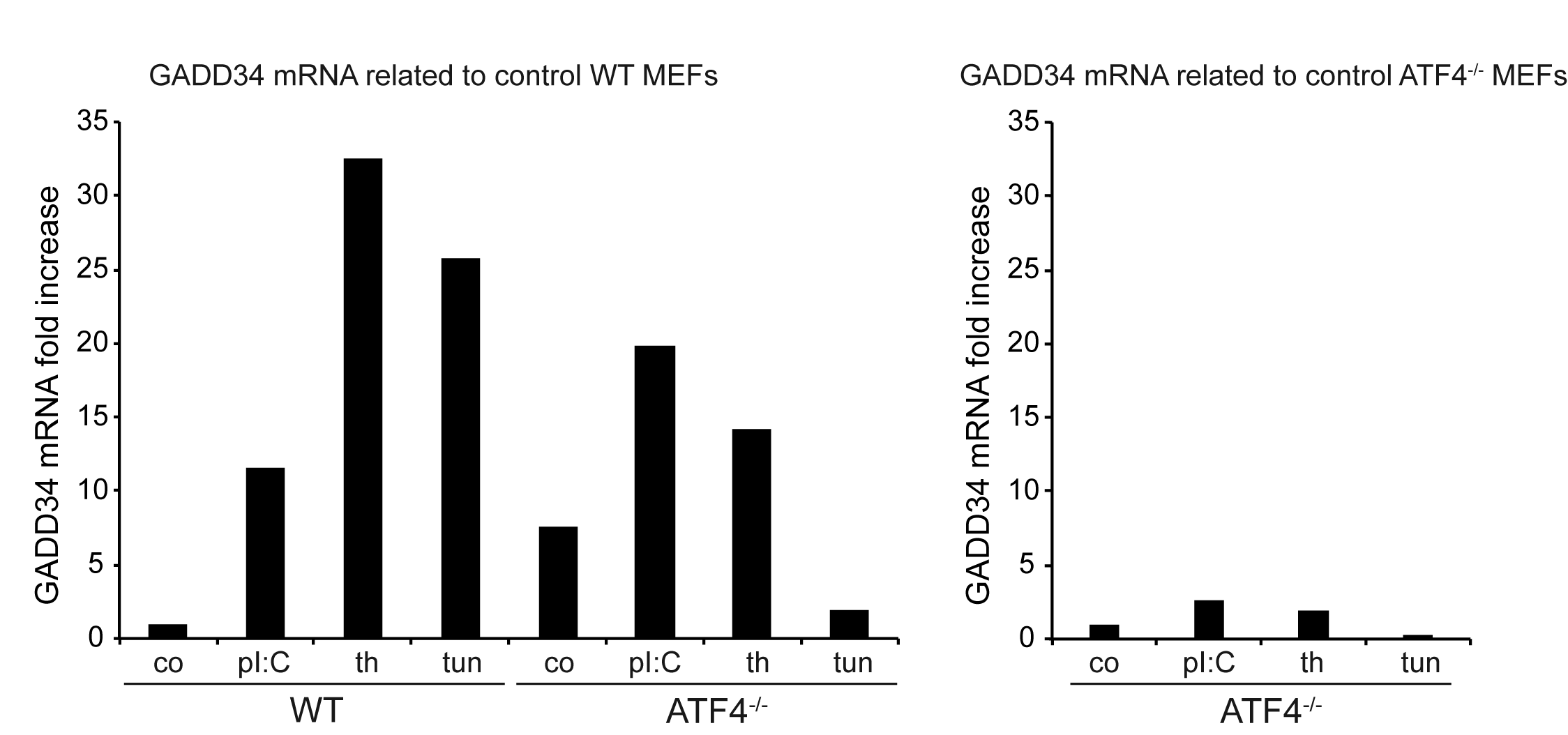

Supplement: Figure S3 — GADD34 mRNA induction in ATF4-deficient MEFs stimulated with cytosolic poly I:C. The levels of GADD34 transcript were determined by qPCR in WT and ATF4−/− cells after 8 h of poly I:C stimulation. Treatment with tunicamycin and thapsigargin were used as positive controls for GADD34 induction. Results are displayed according to both WT internal reference (left) and ATF4−/− internal reference (right). (TIF) [file ppat.1002708.s003.tif]

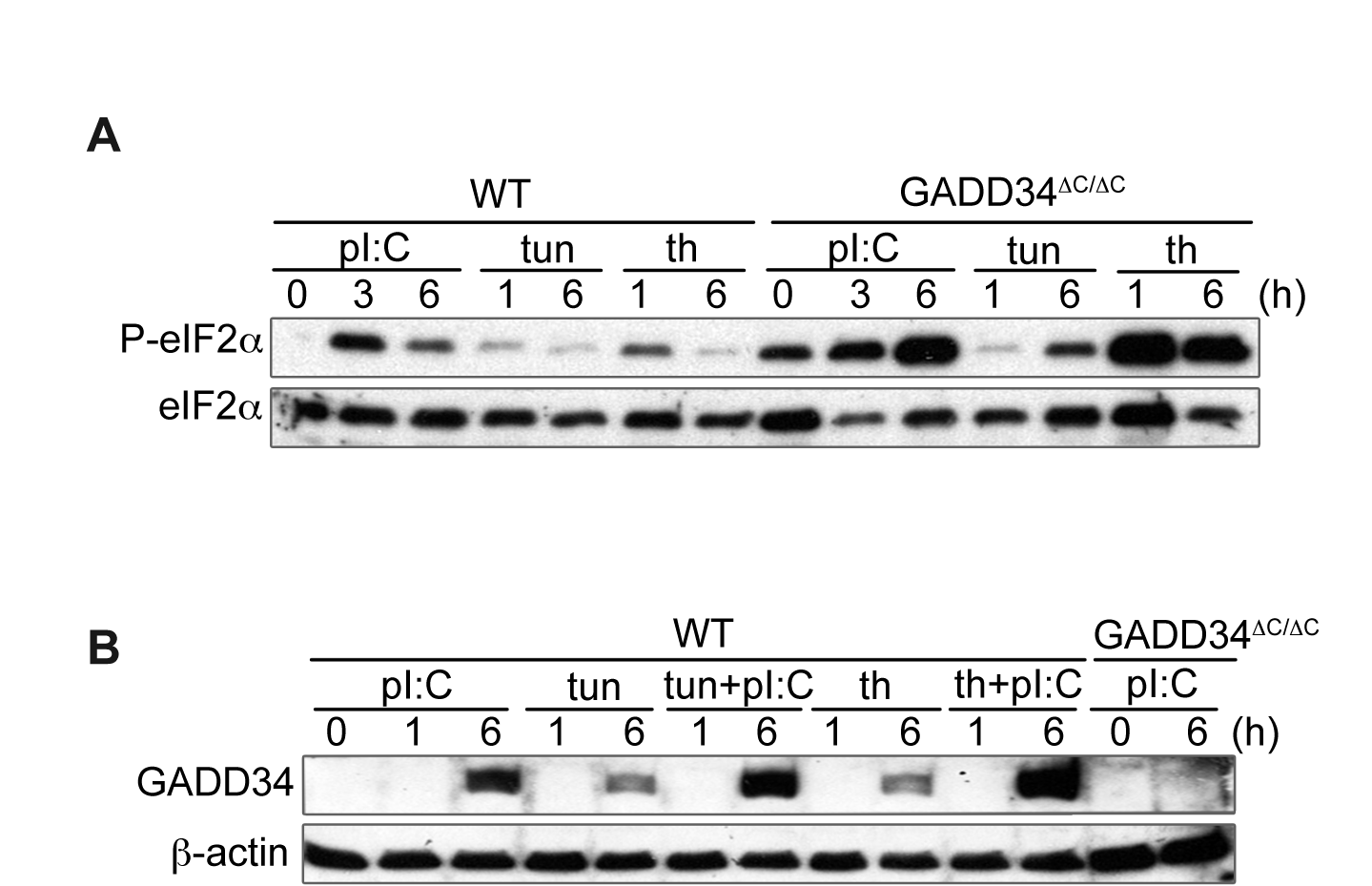

Supplement: Figure S4 — GADD34 mediates eIF2α dephosphorylation in MEFs stimulated with poly I:C. A) Wild-type and GADD34ΔC/ΔC MEFs were treated for the indicated times with poly I:C (pI:C), tunicamycin (tun) or thapsigargin (th) and eIF2α phosphorylation was monitored by immunoblot. B) GADD34 expression was analyzed by immunoblot in samples treated for 1 or 6 hours with poly I:C alone or together with tunicamycin (tun) or thapsigargin (th). Data shown in (A) and (B) are representative of three independent experiments with similar results. (TIF) [file ppat.1002708.s004.tif]

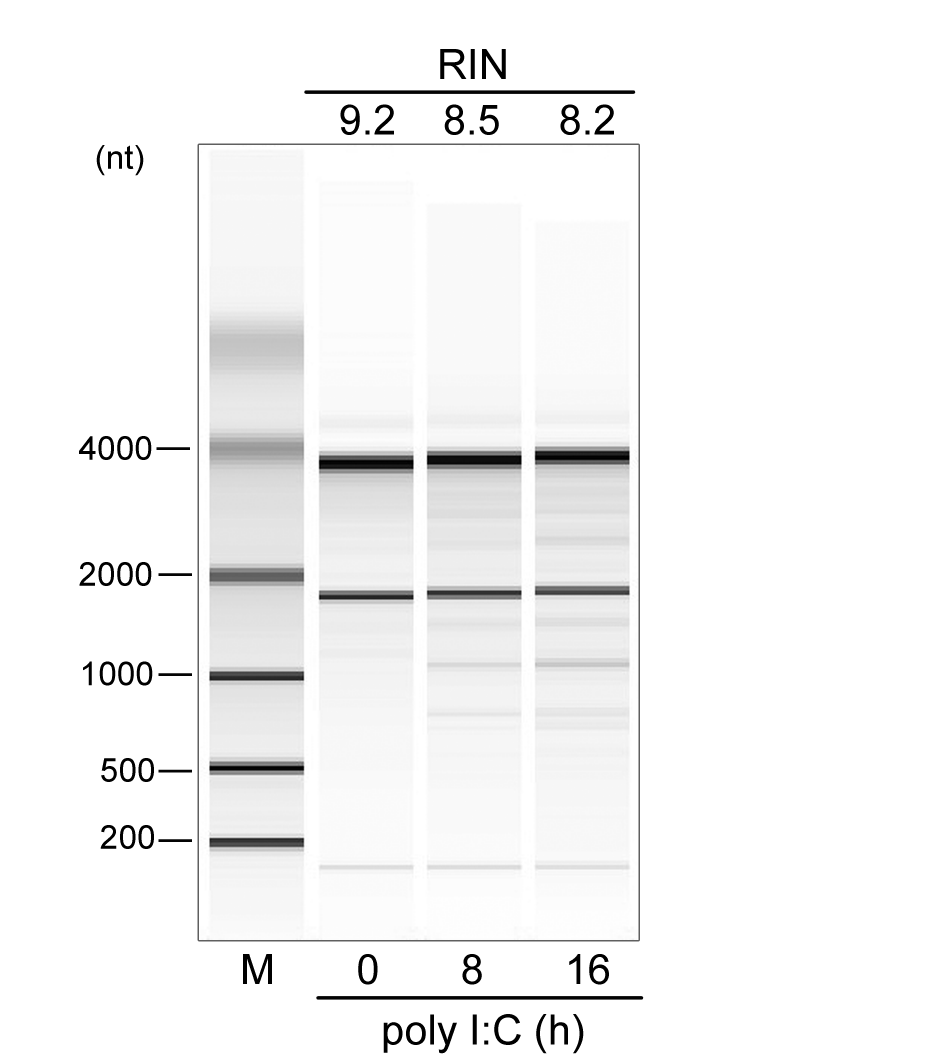

Supplement: Figure S5 — RNA integrity upon poly I:C exposure. WT MEFs were treated with poly I:C for the indicated times and RNA integrity evaluated by capillary electrophoresis (Agilent RNA 6000). RNA Integrity Numbers (RIN) between 8.2 and 9.2 were obtained, indicating a high level of RNA integrity. Data shown are representative of three independent experiments with similar results. (TIF) [file ppat.1002708.s005.tif]

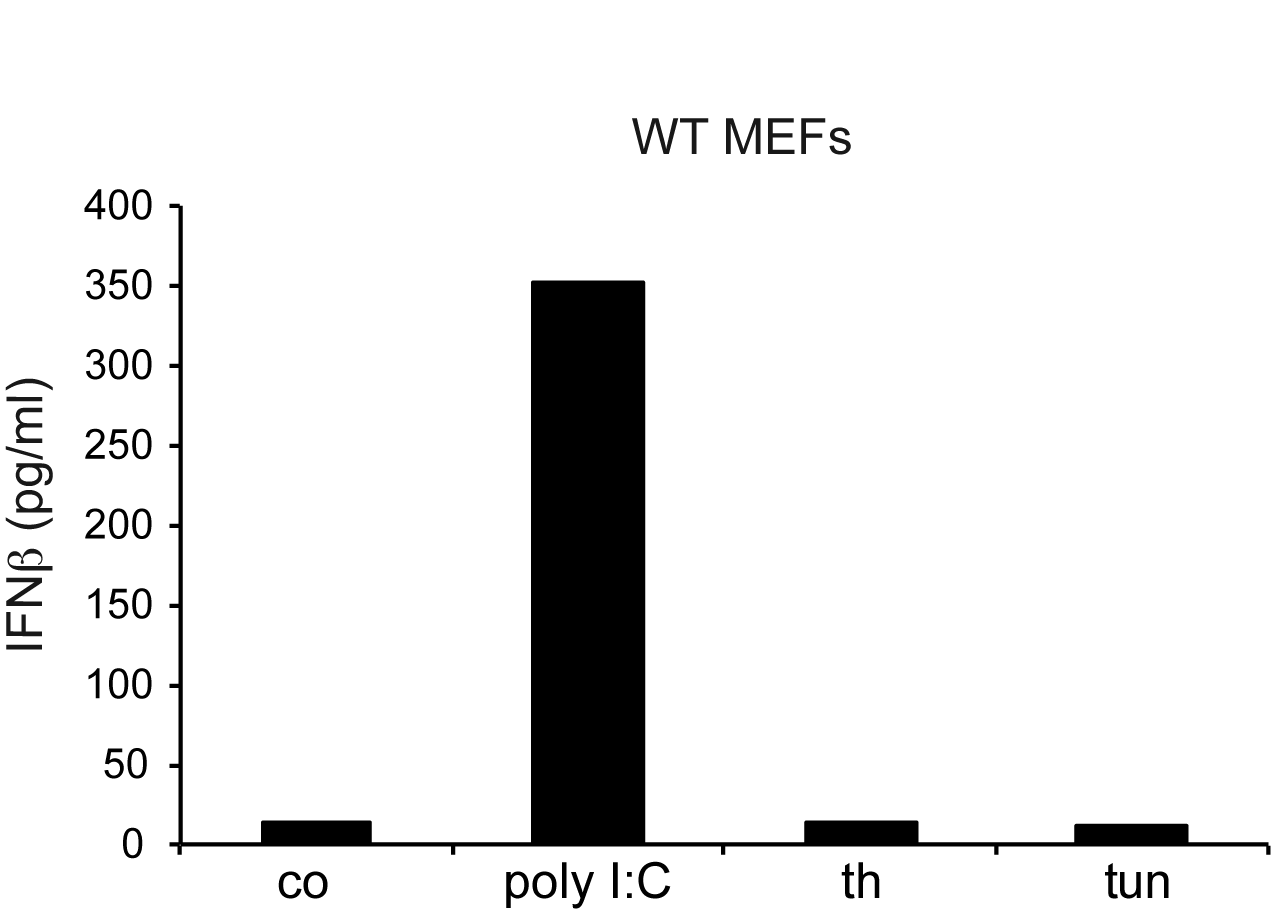

Supplement: Figure S6 — UPR-inducing drugs do not elicit IFN-β production. Cell culture supernatants of murine embryonic fibroblasts were tested for the presence of IFN-β, after treatment with poly I:C (8 h), tunicamycin and thapsigargin (6 h). The results shown are representative of 4 experiments. (TIF) [file ppat.1002708.s006.tif]

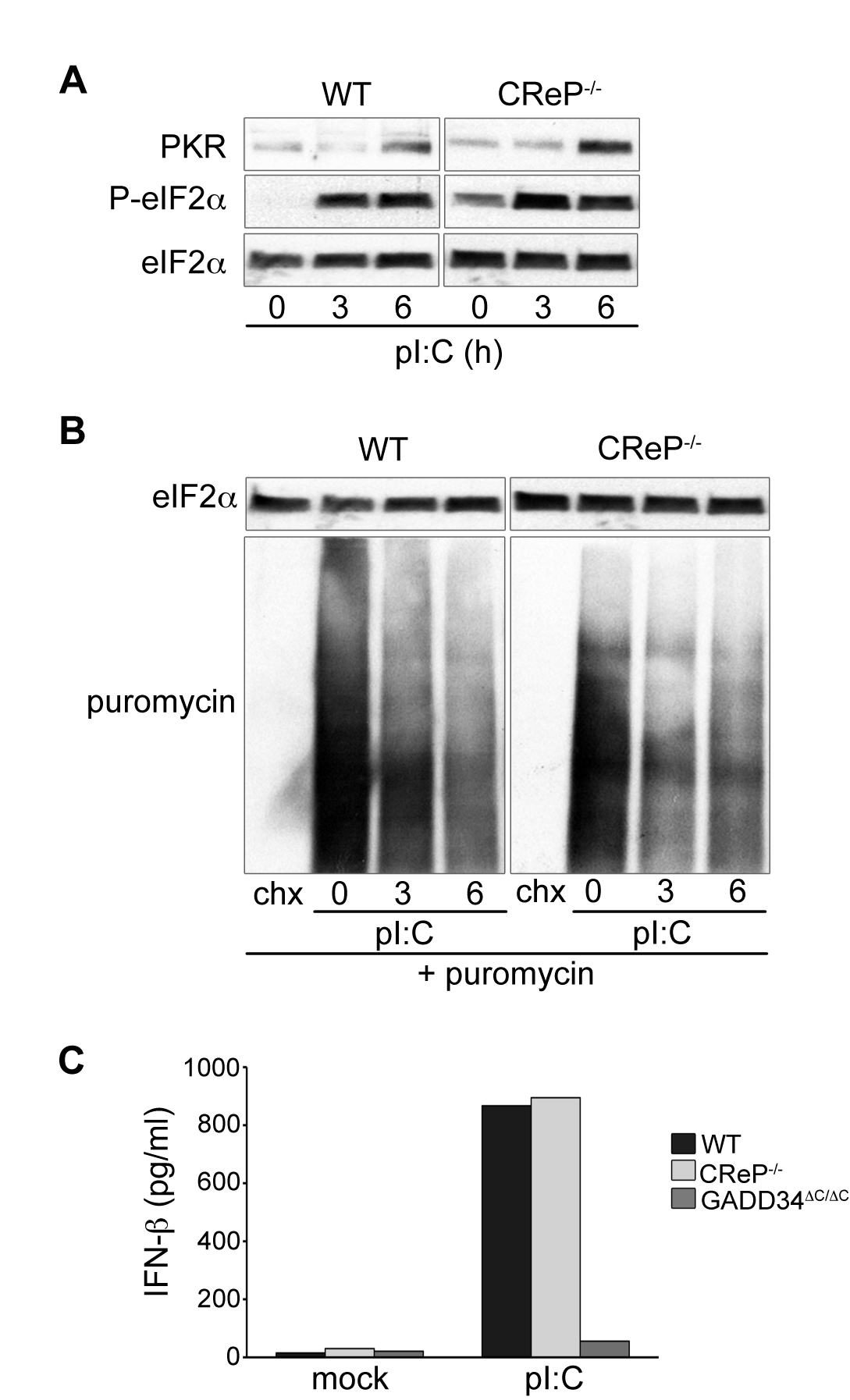

Supplement: Figure S7 — Deletion of the constitutively-expressed PP1 co-factor, CReP, does not impact protein translation and IFN-β production in MEFs. A) WT and CReP−/− MEFs were treated with poly I:C (pI:C) for the indicated times and the levels of P-eIF2α and PKR were analyzed by immunoblot. Although basal levels of P-eIF2α were higher in CReP−/− MEFs, increase of phosphorylation upon poly I:C exposure was similar to the WT. PKR expression upon poly I:C treatment was equivalent in CReP−/− and WT MEFs. B) Protein synthesis was quantified using puromycin labeling followed by immunoblot with the anti-puromycin mAb 12D10. Where indicated, cells were treated with cycloheximide (chx) 5 min before puromycin incorporation. No major differences were found between WT and CReP−/− cells at the level of translation inhibition following poly I:C exposure. C) IFN-β quantification in cell culture supernatants after 8 h of poly I:C (pI:C) treatment. Data shown in this figure are representative of two independent experiments with similar results. (TIF) [file ppat.1002708.s007.tif]

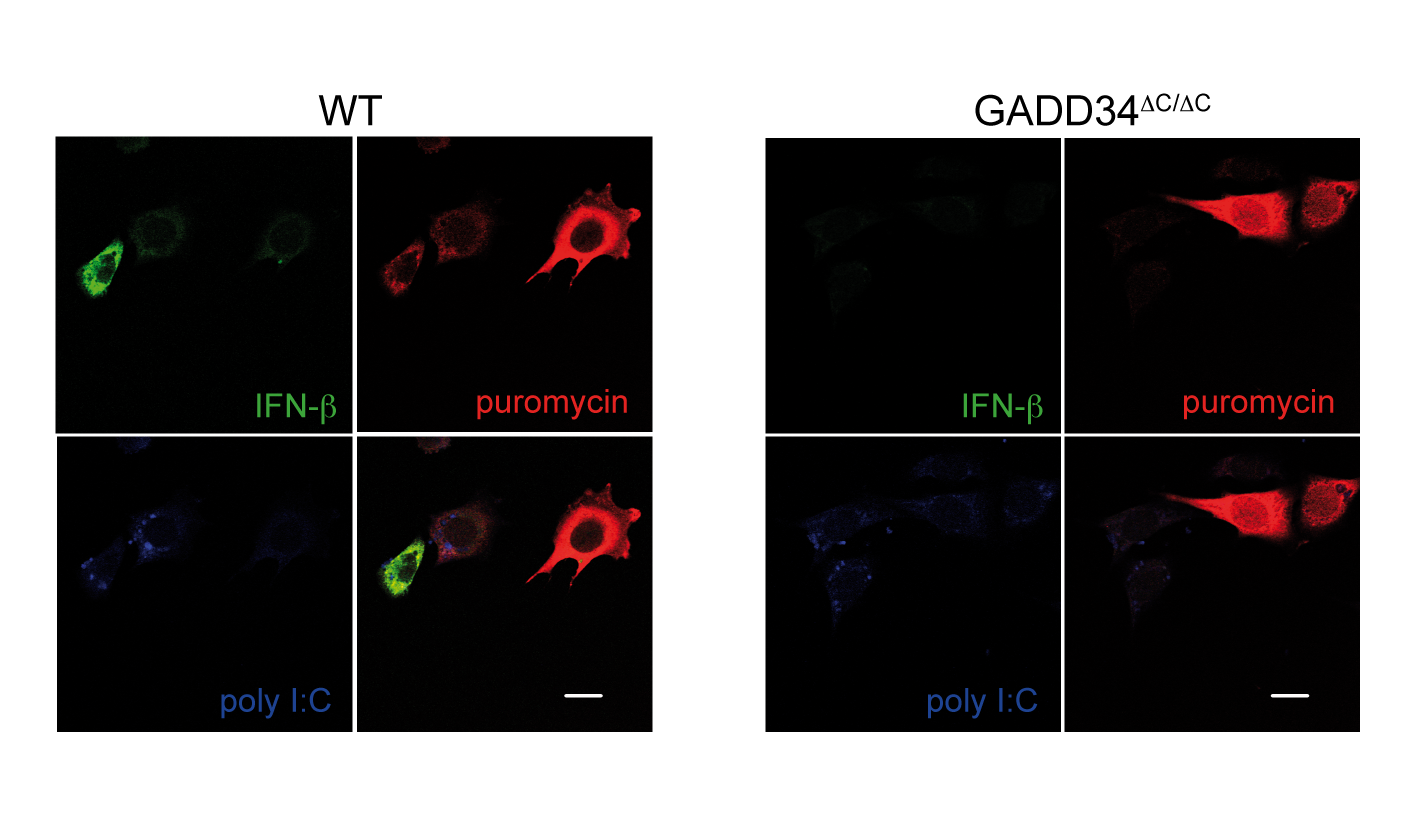

Supplement: Figure S8 — GADD34 is necessary for IFN-β production in response to poly I:C stimulation. WT and GADD34ΔC/ΔC MEFs were treated with poly I:C for 8 h and labeled with puromycin for the last 10 min. Immunofluorescence staining for intracellular IFN-β, puromycin (red) and dsRNA (poly I:C, blue) was performed and samples were imaged by confocal microscopy. Scale bar, 10 µm. Data shown are representative of three independent experiments with similar results. (TIF) [file ppat.1002708.s008.tif]

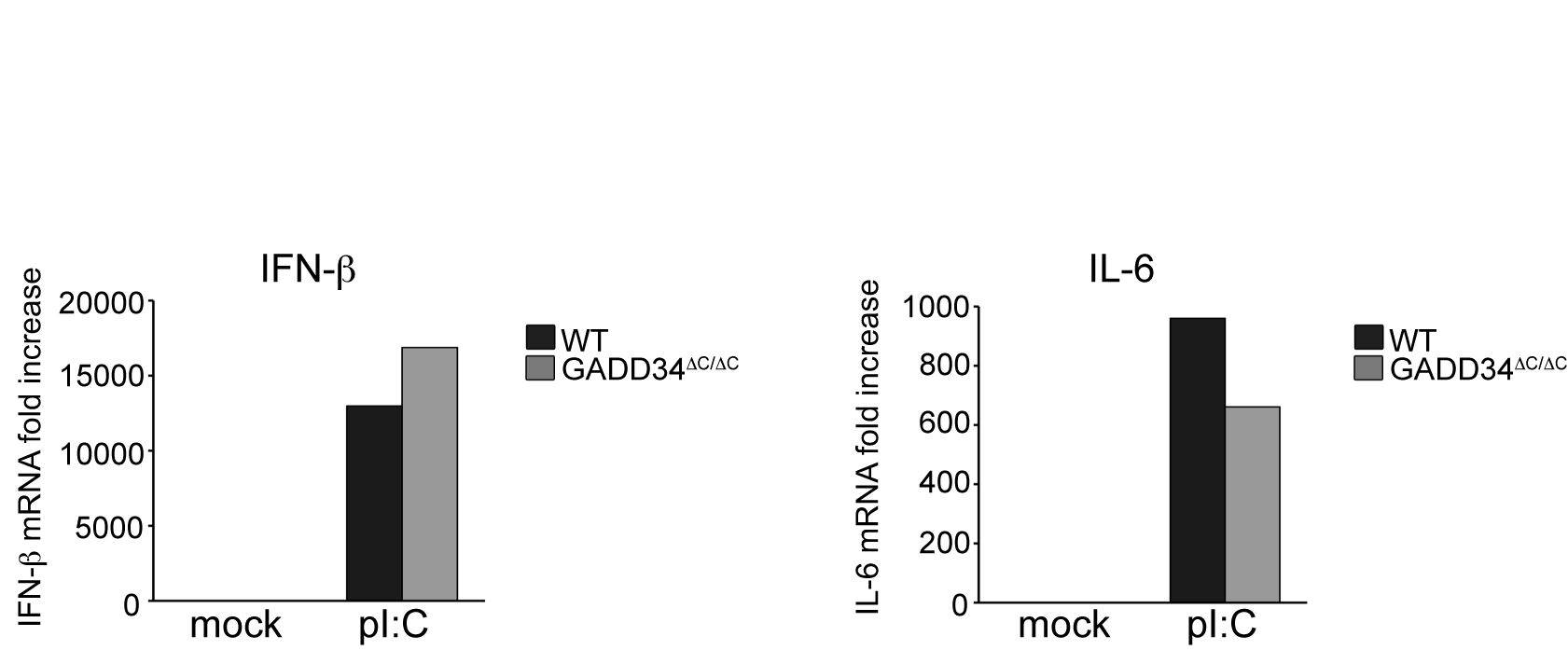

Supplement: Figure S9 — IFN-β and IL-6 polyA+ mRNAs are induced equally in WT and GADD34ΔC/ΔC MEFs in response to dsRNA. WT and GADD34ΔC/ΔC MEFs were treated for 6 h with poly I:C, total RNA extracted and poly A+ mRNAs purified on an oligo-dT column. Quantitative PCR was performed after reverse transcription. Data shown are representative of two independent experiments with similar results. (TIF) [file ppat.1002708.s009.tif]

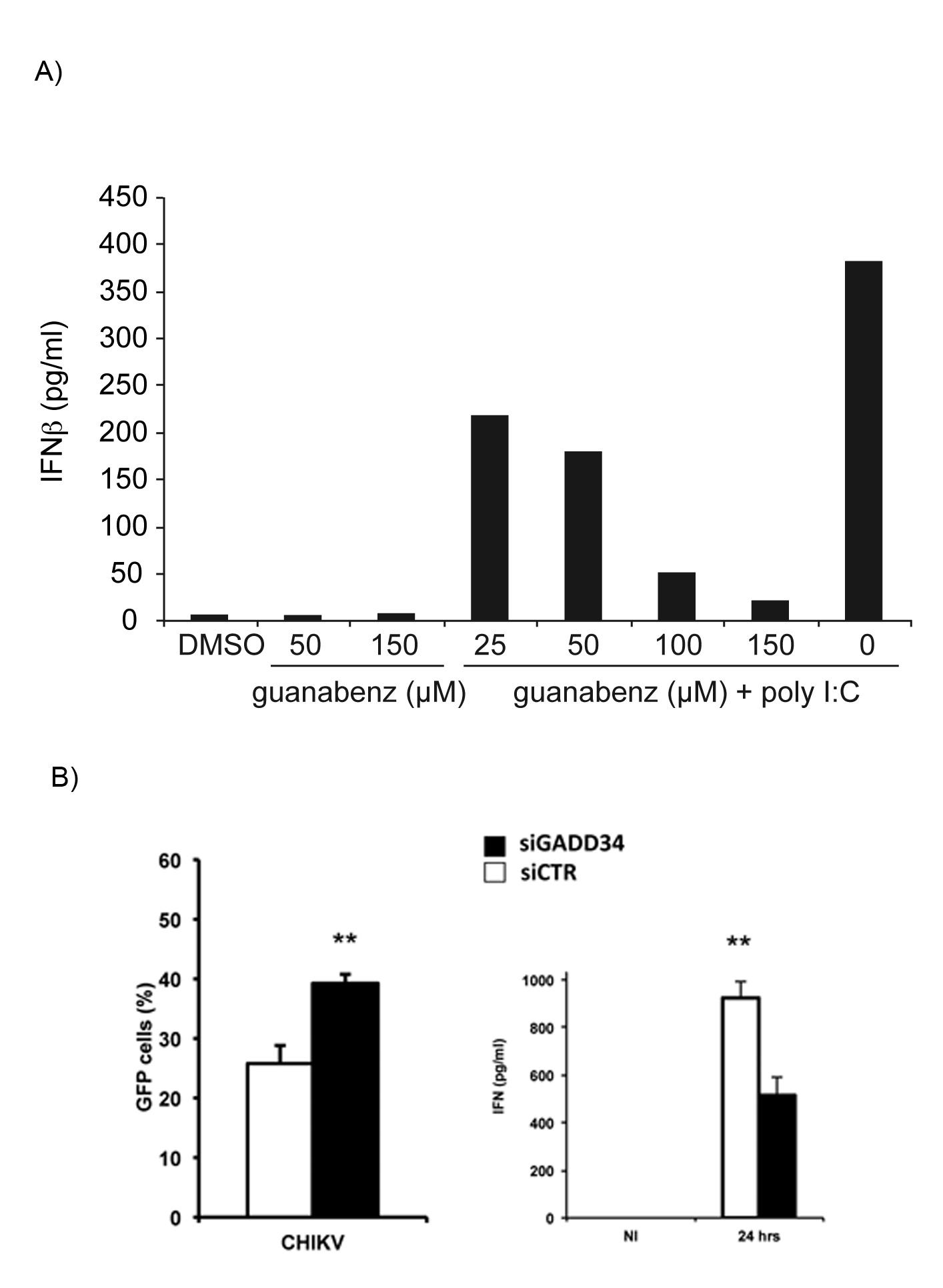

Supplement: Figure S10 — Specific inhibition of GADD34 with guanabenz or by RNAi decreases IFN-β production. A) WT MEFs were treated with different doses of guanabenz (or DMSO as control) during 2 hours before being stimulated with poly I:C for 8 hours in the presence or absence of guanabenz. IFN-β levels were monitored in cell culture supernatants after the treatments. Guanabenz decreased IFN-β levels in a dose-dependent fashion. Data shown is representative of three independent experiments with similar results. B) MEFs treated with con and GADD34 siRNAs were infected with CHIKV-GFP for a period of 24 h. The percentage of infected GFP positive cells and resulting IFN-ß production were analyzed. (TIF) [file ppat.1002708.s010.tif]
